# Reconstitution of Mycobacterium marinum Nonhomologous DNA End Joining Pathway in *Leishmania*

**DOI:** 10.1128/msphere.00156-22

**Published:** 2022-06-13

**Authors:** Wen-Wei Zhang, Douglas G. Wright, Lynn Harrison, Greg Matlashewski

**Affiliations:** a Department of Microbiology and Immunology, McGill University, Montreal, Quebec, Canada; b Department of Molecular and Cellular Physiology, Louisiana State University Health Sciences Center, Shreveport, Louisiana, USA; University at Buffalo

**Keywords:** *Leishmania donovani*, CRISPR-Cas9, gene targeting, gene deletion, double-strand DNA break, DSB repair, single-strand annealing, SSA, nonhomologous end joining, NHEJ, Ku, Ku70, Ku80, telomere maintenance, *Mycobacterium marinum*, Ligase D

## Abstract

In mammalian cells, DNA double-strand breaks (DSBs) are mainly repaired by nonhomologous end joining (NHEJ) pathway. Ku (a heterodimer formed by Ku70 and Ku80 proteins) and DNA ligase IV are the core NHEJ factors. Ku could also be involved in other cellular processes, including telomere length regulation, DNA replication, transcription, and translation control. *Leishmania*, an early branching eukaryote and the causative agent of leishmaniasis, has no functional NHEJ pathway due to its lack of DNA ligase IV and other NHEJ factors but retains Ku70 and Ku80 proteins. In this study, we generated Leishmania donovani Ku70 disruption mutants and Ku70 and Ku80 double gene (Ku70/80) disruption mutants. We found that *Leishmania* Ku is still involved in DSB repair, possibly through its binding to DNA ends to block and slowdown 5′ end resections and Ku-Ku or other protein interactions. Depending on location of a DSB between the direct repeat genomic sequences, *Leishmania* Ku could have an inhibiting effect, no effect or a promoting effect on the DSB repair mediated by single strand annealing (SSA), the most frequently used DSB repair pathway in *Leishmania*. Ku70/80 proteins are also required for the healthy proliferation of *Leishmania* cells. Interestingly, unlike in Trypanosoma brucei and L. mexicana, Ku70/80 proteins are dispensable for maintaining the normal lengths of telomeres in L. donovani. We also show it is possible to reconstitute the two components (Ku and Ligase D) NHEJ pathway derived from Mycobacterium marinum in *Leishmania*. This improved DSB repair fidelity and efficiency in *Leishmania* and sets up an example that the bacterial NHEJ pathway can be successfully reconstructed in an NHEJ-deficient eukaryotic parasite.

**IMPORTANCE** Nonhomologous end joining (NHEJ) is the most efficient double-stranded DNA break (DSB) repair pathway in mammalian cells. In contrast, the protozoan parasite *Leishmania* has no functional NHEJ pathway but retains the core NHEJ factors of Ku70 and Ku80 proteins. In this study, we found that *Leishmania* Ku heterodimers are still participating in DSB repair possibly through blocking 5′ end resections and Ku-Ku protein interactions. Depending on the DSB location, Ku could have an inhibiting or promoting effect on DSB repair mediated by the single-strand annealing repair pathway. Ku is also required for the normal growth of the parasite but surprisingly dispensable for maintaining the telomere lengths. Further, we show it is possible to introduce Mycobacterium marinum NHEJ pathway into *Leishmania*. Understanding DSB repair mechanisms of *Leishmania* may improve the CRISPR gene targeting specificity and efficiency and help identify new drug targets for this important human parasite.

## INTRODUCTION

In order to maintain genome integrity and survive, living cells must repair DNA double-strand breaks (DSBs) generated by various endogenous or exogenous stresses, including CRISPR gene targeting. Depending on the organism, cell type, and cell cycle phase, DSBs can be repaired through one of the following ends joining pathways, namely, nonhomologous end joining (NHEJ), microhomology-mediated end joining (MMEJ), single-strand annealing (SSA), and homologous recombination (HR) (1–3). Although those pathways may share some repair proteins and steps, each of those pathways requires specific repair factors. The presence of those specific factors, their relative activity, and availability of homologous repair sequences determine the repair pathway choice for a DSB. Due to differences of repair mechanisms, those pathways can produce very different repair outcomes from faithful repair to extensive deletions (1–3).

In human cells, DSBs are mainly repaired by NHEJ (>70% of repair events) and HR (<30% of repair events, mainly during S and G_2_ cell cycle phases when the sister chromatid is available for recombination) (4–6). The Ku70/80 heterodimer (Ku) formed by the abundant Ku70 and Ku80 nuclear proteins plays a central role in NHEJ. Ku, a ring-like structure, has very high affinity for DSB ends. Ku rapidly recognizes and threads onto broken DNA ends (within seconds) in the cell nucleus. Ku subsequently recruits other NHEJ effectors, including DNA-PKcs (DNA-dependent protein kinase catalytic subunit), nucleases, polymerases, and the DNA Ligase IV complex which form a DNA synapse to bridge the ends. After minor processing of the ends by nucleases or polymerases to expose short complementary sequences (1 to 4 nucleotides [nt]), the ligatable termini are then rejoined by Ligase IV, usually within 30 min after DSB. NHEJ typically attempts to repair DSBs first, often resulting in small deletions or insertions and is much faster than HR (7 h or longer is required for HR) (4–8).

If NHEJ fails to repair the DSB, the Ku heterodimer rings will be displaced from the DNA ends by the Mre11, Rad50, and Nbs1 (MRN) complex which initiates 5′-end resections to generate 3′-single-stranded DNA (ssDNA) overhangs suitable for repair with MMEJ, SSA, or HR pathways (9–13). If the DSB occurs in cell cycle S and G2 phases, HR is active as sister chromatids are present in the cell. The nucleoprotein filaments formed from the 3′-ssDNA overhangs, replication protein A (RPA), and Rad51 recombinase protein will search for and invade the adjacent homology sequence in the sister chromatid as the HR repair template. As a result, DNA is often repaired faithfully by HR with no insertion or deletions (5). Because Ku blocks 5′-end resections, MMEJ is *per se* inefficient and SSA requires the existence of long repeat sequences flanking the DSB and extensive resections, MMEJ and SSA are rarely used for DSB repair in mammalian cells. They are the backup pathways when the NHEJ and HR pathways are absent or interrupted, such as in some cancer cells (10–15). MMEJ uses the microhomology sequences (5 to 25 nt) in the 3′-overhangs to anneal the two single-stranded DNA and causes deletion between the microhomology sequences. DNA polymerase θ helps anneal the microhomology sequences and plays a central role in MMEJ (12, 13). SSA mediated repair can take place if the DSBs occur between two direct repeat sequences (>26 bp). SSA usually requires extensive resections to reach the homologous repeat sequences flanking the DSB. With the assistance of Rad52 and other repair proteins, the single-strand 3′-overhangs are annealed between the complementary sequences of direct repeats located on opposite sides of the DSB, followed by the nucleolytic removal of remaining tails, DNA synthesis to fill in gaps, and ligation. Depending on the distance between the repeats, SSA often results in large deletion mutations and causes the most severe damage to the genome (15).

Unlike prokaryotic Escherichia coli which has only the HR DSB repair pathway, mycobacteria possess HR as well as NHEJ and SSA pathways (16). However, NHEJ in mycobacteria such as Mycobacterium tuberculosis and Mycobacterium marinum is mainly facilitated by two proteins, Ku and Ligase D (17–19). *In vitro* and *in vivo* studies have shown that mycobacterial Ku forms a homodimer which has high affinity for DNA ends and interacts physically with Ligase D and promotes its ligation activity (17, 20). Unlike Ligase IV in mammalian cells, Ligase D also contains nucleotidyl transferase activities, including gap-filling polymerase, terminal transferase, and primase, and the 3′- to 5′-exonuclease activity. Thus, Ligase D from mycobacteria can carry out DNA ends processing and join incompatible DSB ends (17). It has been demonstrated that the mycobacterial NHEJ pathway could be successfully reconstituted to repair DSBs in yeast and in Escherichia coli by introducing the Ku and Ligase D proteins (17–19).

*Leishmania* protozoan, an early branching eukaryote, is the causative agent of human leishmaniasis, one of the major neglected tropical diseases. Visceral leishmaniasis, caused by L. donovani is the second most deadly parasitic infection after malaria. There are no approved vaccines for leishmaniasis. Treatment of Leishmania infection relies on chemical drugs with side effects (21). Contrary to mammalian cells, *Leishmania* lacks the highly efficient NHEJ pathway due to absence of several key NHEJ factors, including DNA-PKcs, Ligase IV, and XRCC4, though, it still retains the core NHEJ factor Ku70 and Ku80 proteins (22–24). Like in many other organisms, homologous chromosomes are rarely used as the HR template for repairing DSB (25). Instead, *Leishmania* mainly relies on SSA (>95% of events if the direct repeat sequences flanking the DSBs exist) and MMEJ to repair DSBs, which involve extensive resections, prolonged repair time, and often result in large sequence deletions (up to 77 kb was detected) and frequently fail (26, 27). It was estimated that more than half of the DSBs could not be repaired in *Leishmania*, leading to cell death (25, 26, 28). Because of lack of a functional NHEJ DSB repair pathway, compared with other organisms, CRISPR gene targeting is not very efficient in *Leishmania* and antibiotic selection marker encoding donor DNAs (a kind of SSA mediated repair) are often required to isolate the CRISPR edited mutants (28–31).

As indicated above, the Ku dimer has strong affinity for DSB ends (7). If NHEJ could not be completed due to lack of other key NHEJ factors, Ku70/80 binding could block the 5′-end resections to prevent or slowdown other DSB repair pathways which rely on using the 3′-single-stranded DNA overhangs (32–36). For example, it has been recently reported that Ku70/80 heterodimer suppresses MMEJ in Ligase IV-deficient progenitor B cells, deletion of Ku70 in this B cell line significantly increases MMEJ (33). In addition, Ku has also been shown to be involved in other cellular processes such as telomere maintenance, DNA replication, transcription, and translation control (7, 8, 37–45). In this study, to investigate whether *Leishmania* Ku70 and Ku80 proteins actively participate in DSB repair in the absence of key NHEJ factors Ligase IV and XRCC4, we generated Leishmania donovani Ku70 null mutants and Ku70/80 double gene null mutants. It was observed that depending on location of a DSB between the direct repeat sequences, L. donovani Ku70/80 heterodimer could inhibit, have no effect, or assist DSB repair by SSA. In addition, Ku70/80 proteins are required for the normal healthy cell proliferation. However, unlike in other organisms (43–45), the normal lengths of the telomeres are maintained in Ku-deficient L. donovani cells. We also introduced Mycobacterium marinum two components (Ku and Ligase D) NHEJ pathway into *Leishmania*. This improved CRISPR gene targeting specificity and efficiency in *Leishmania* and represents the first example that the bacterial NHEJ pathway can be successfully reconstructed for DSB repair in an NHEJ-deficient eukaryotic parasite.

## RESULTS AND DISCUSSION

### Generation of L. donovani Ku70 null mutants and Ku70 and Ku80 double null mutants.

**FIG 1:**
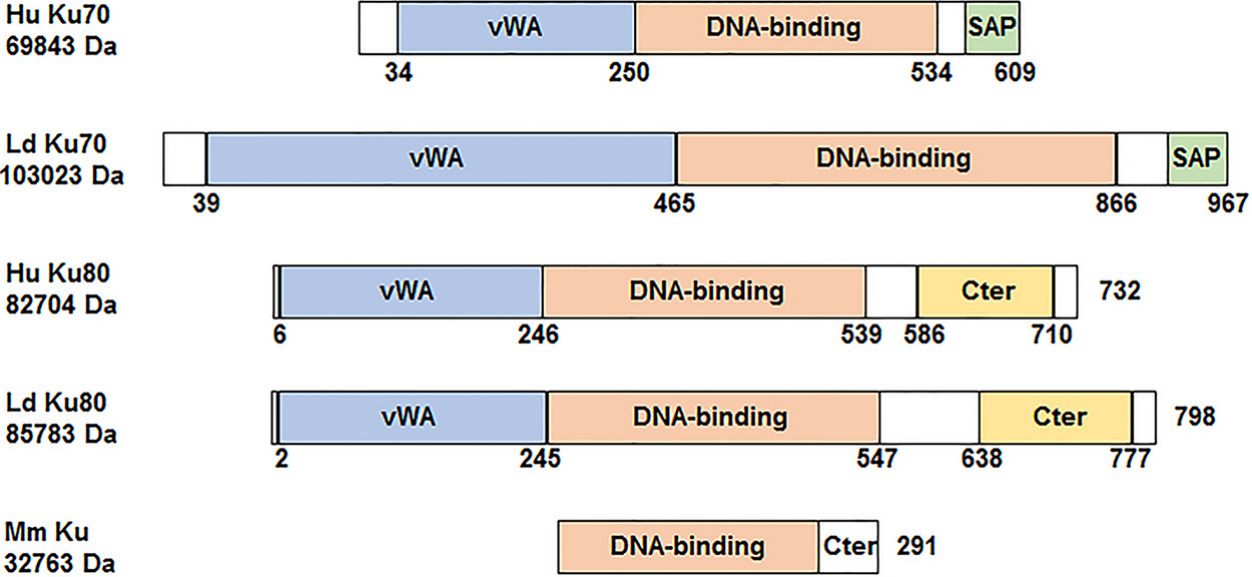
Schematics of Ku70 and Ku80 proteins of human and Leishmania donovani and Ku protein of Mycobacterium marinum. Like human Ku proteins, *Leishmania* Ku proteins also possess the Ku von Willebrand A (vWA) domain, DNA-binding domain, and SAF-A/B, Acinus, and PIAS (SAP) or C-terminal domain (Cter). While Ld Ku80 is only slightly larger than human Ku80, Ld Ku70 contains 967 amino acids and has a molecular weight of 103 kDa which is 30 kDa larger than Hu Ku70. Unlike eukaryotic cells, the prokaryote Mycobacterium marinum has only a single small Ku protein of 33 kDa mainly composed of the DNA binding domain and a short C-terminal domain (Cter). The Mm Ku Cter domain is involved in binding and activating Mm Ligase D and may also has some nonspecific DNA binding activity.

As noted in the Introduction, *Leishmania* retains Ku proteins but lacks other key NHEJ effectors, including DNA Ligase IV, DNA PKcs, and XRCC 4 (24). It was therefore interesting to compare *Leishmania* Ku to Ku proteins of human and mycobacteria that possess a functional NHEJ. L. donovani (Ld) Ku70 and Ku80 proteins retain the conserved domains present in human Ku proteins, including the N-terminal von Willebrand A domain, the central core DNA-binding domain and a divergent C-terminal region. Ld Ku70, however, has about 27% amino acid identity with human Ku70 and is larger containing 967 amino acids with molecular weight of 103,023 Dalton (Fig. 1; Fig. S1). Ld Ku80 protein has a similar size (798 amino acids) as human Ku80 protein (732 amino acids) and they share 22% amino acid sequence identity (Fig. 1; Fig. S1). Like human Ku proteins, Ld Ku70 and Ku80 proteins also share some sequence similarity with 21% amino acid identity over 660 amino acids alignment (Fig. S1). In contrast to eukaryotes, mycobacteria such as *Mycobacteria marinum* possess only a single small Ku protein of 291 amino acids which forms a Ku homodimer for binding DNA ends and NHEJ repair (19). Interestingly, Ld Ku70 and Ku80 also share 20% to 24% amino acid identities in their DNA binding domain with M. marinum Ku used in this study (Fig. 1; Fig. S1; see below).

10.1128/msphere.00156-22.1 FIG S1 Amino acids alignments of Ku70 and Ku80 proteins of human and Leishmania donovani and Ku protein of Mycobacterium marinum. (A) Amino acids alignment of human Ku70 and Leishmania donovani Ku70. (B) Amino acids alignment of human Ku80 and Leishmania donovani Ku80. (C) Amino acids alignment of Leishmania donovani Ku70 and Ku80. (D) Amino acids alignment of Leishmania donovani Ku70 and Mycobacterium marinum Ku. (E) Amino acids alignment of Leishmania donovani Ku80 and Mycobacterium marinum Ku. Download FIG S1, DOCX file, 0.1 MB. Copyright © 2022 Zhang et al. 2022 Zhang et al. https://creativecommons.org/licenses/by/4.0/ This content is distributed under the terms of the Creative Commons Attribution 4.0 International license.

**FIG 2:**
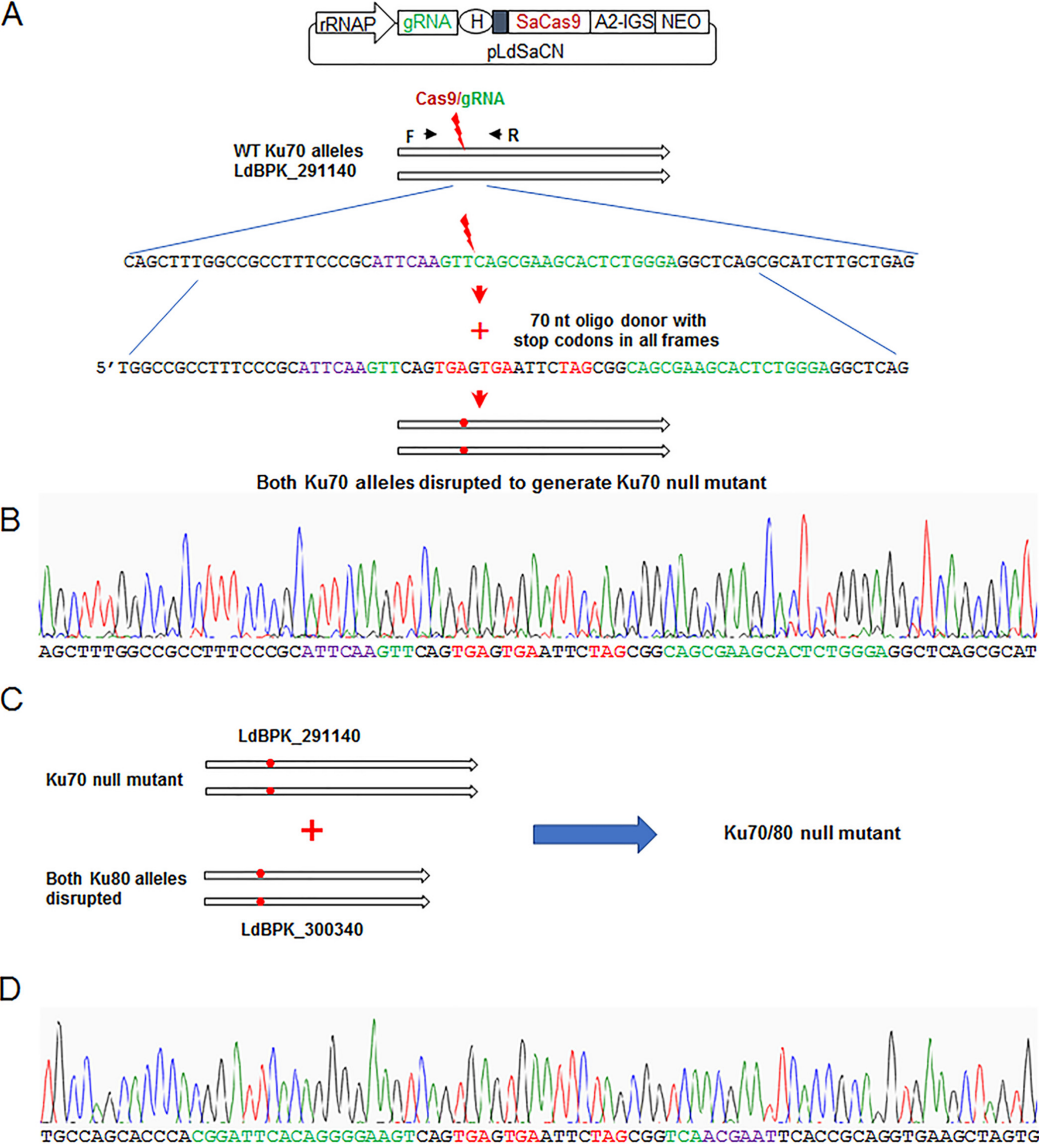
Generation of L. donovani Ku70 gene disruption mutant and Ku70 and Ku80 double gene disruption mutant. (A) The CRISPR strategy used to disrupt both alleles of Ld Ku70 genes with stop codons containing oligo donors. pLdSaCN CRISPR vector expresses Staphylococcus aureus Cas9 and a gRNA targeting Ld Ku70 gene. The gRNA targeting site is highlighted as green, the SaCas9 PAM site highlighted as purple. (B) Chromatogram showing both Ld Ku70 gene alleles were disrupted as planned with the stop codons containing oligo donors. (C) The same CRISPR strategy was used to further disrupt both alleles of Ld Ku80 gene in the Ld Ku70 disruption mutant to generate the Ku70 and Ku80 double gene disruption mutant (please see details in Fig. S2). (D) Chromatogram showing both Ld Ku80 gene alleles were disrupted as planned with the stop codons containing oligo donors.

To investigate whether *Leishmania* Ku proteins are involved in DSB repair, we generated L. donovani Ku null mutants using the CRISPR method we previously described (46). To facilitate generating double gene knock out mutants and save the antibiotic selection markers for succeeding studies, oligonucleotides with stop codons were used as donors (repair templates) following DSB to disrupt both L. donovani Ku70 (LdBPK_291140) and Ku80 (LdBPK_300340) genes (Fig. 2A). To generate L. donovani Ku70 null mutants [Ku70(-)], the *Leishmania* CRISPR plasmid pLdSaCN-Ld291140, expressing neomycin (G418) resistance gene, SaCas9 nuclease and a gRNA targeting the middle in first half of the Ku70 gene (Fig. 2A) was transfected into L. donovani promastigotes. The G418 resistant cells were subsequently transfected with a 70 nt oligonucleotides donor with stop codons in all frames. PCR screening with a donor specific primer and DNA sequencing confirmed that both alleles of L. donovani Ku70 gene were disrupted as planned with the stop codons containing oligo donor (Fig. 2B). Likewise, to generate L. donovani Ku70 and Ku80 double gene knock out mutants [Ku70/80(-)], Ku70 null mutants [Ku70(-)] were retransfected with CRISPR plasmid pLdSaCH-Ld300340 which expresses hygromycin resistance gene, SaCas9 nuclease and a gRNA targeting the first half of Ku80 gene (Fig. 2C; Fig. S2A). The hygromycin resistant cells were then transfected with the stop codons containing oligonucleotides. PCR screening and DNA sequencing revealed that both alleles of L. donovani Ku80 gene were disrupted with the introduced stop codons (Fig. 2D; Fig. S2B). The CRISPR plasmids pLdSaCN and pLdSaCH were later removed from the Ku70(-) and Ku70/80(-) mutants by culturing in G418- and Hygromycin-free medium. Thus, with CRISPR gene editing, we generated the Ku70 single gene knock out mutants and Ku70/80 double gene knock out mutants with no remaining antibiotic selection markers. A total four Ku70(-) clones and three Ku70/80(-) clones were isolated. To avoid the potential clonal variation effect, all the Ku70(-) and Ku70/80(-) clones were pooled for subsequent characterization studies (see below).

10.1128/msphere.00156-22.2 FIG S2 Generation of L. donovani Ku70 and Ku80 double gene disruption mutant. (A) The CRISPR strategy used to disrupt both alleles of Ld Ku80 genes with stop codons containing oligo donors in Ku70 gene disruption mutant. pLdSaCH CRISPR vector expresses Staphylococcus aureus Cas9 and a gRNA targeting Ld Ku80 gene. The gRNA targeting site is highlighted as green, the SaCas9 PAM site highlighted as purple. (B) Chromatogram showing both Ld Ku80 gene alleles were disrupted as planned with the stop codons containing oligo donors. Download FIG S2, TIF file, 1.4 MB. Copyright © 2022 Zhang et al. 2022 Zhang et al. https://creativecommons.org/licenses/by/4.0/ This content is distributed under the terms of the Creative Commons Attribution 4.0 International license.

### *Leishmania* Ku proteins bind DSB ends and influence DSB repair.

**FIG 3:**
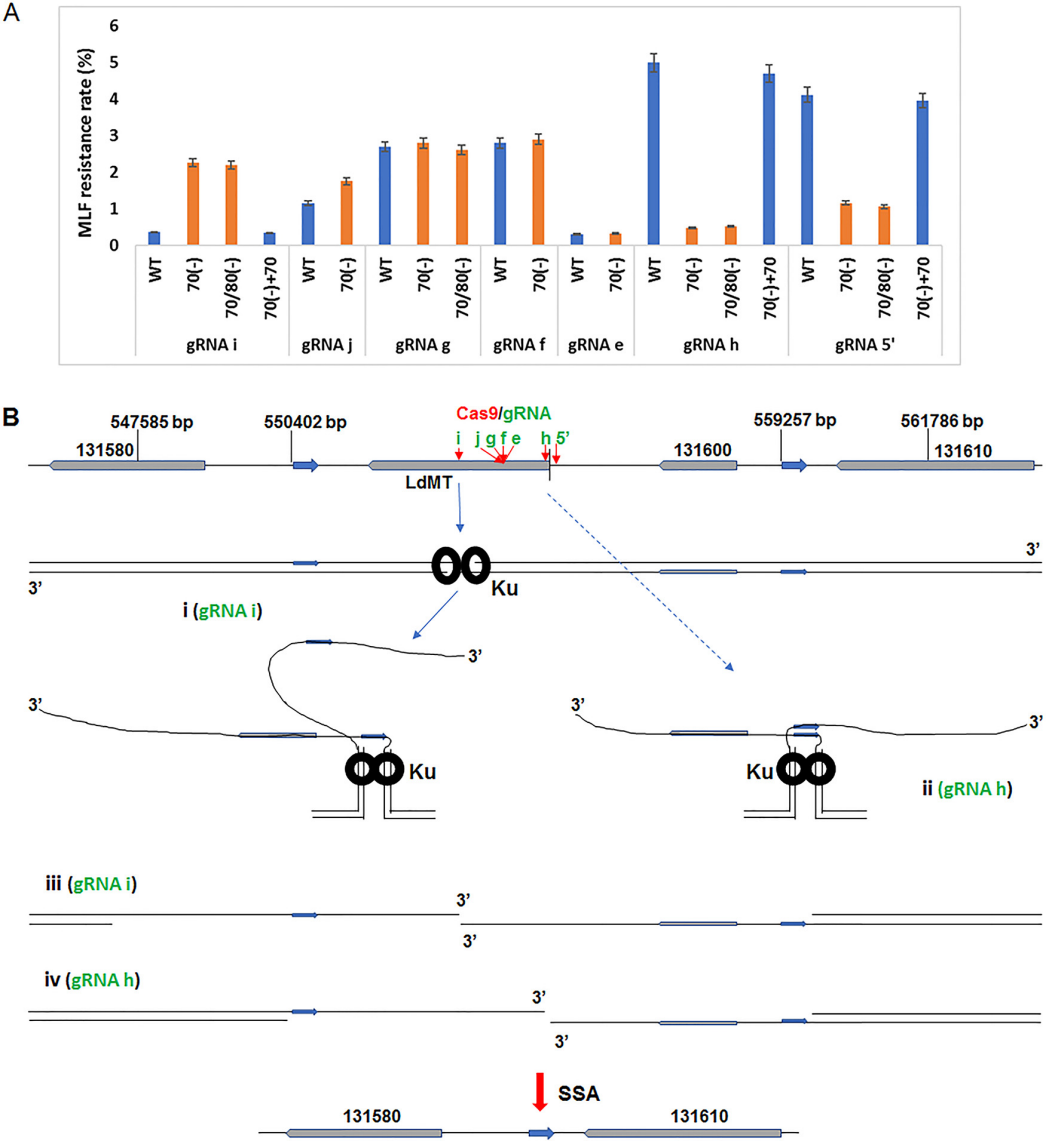
*Leishmania* Ku is still involved in DSB repair. (A) Comparison of MLF resistant rates of WT, Ld Ku70- and Ku70/80-deficient *Leishmania* cells 7-weeks posttransfection of CRISPR vectors expressing various gRNAs targeting *MT* gene. As explained in text and (B), MLF resistant rate also represents the frequency of successful repairs of DSBs generated on both alleles of *MT* gene following CRISPR targeting. The MLF resistance rates shown are mean value obtained from three experiments with standard error of the mean, SEM. (B) A speculative model explaining why the DSB repair efficiencies vary among different gRNAs in WT and Ku-deficient *Leishmania* cells. L. donovani *MT* gene (Ld131590) together with Ld131600 gene are flanked by two 460 bp direct repeats (blue arrows). The positions of those genes and direct repeats in L. donovani chromosome 13 are indicated. The geomatic center between the two repeats is marked with a vertical black line. The seven gRNA targeting sites in *MT* gene are marked with red arrows. Note, the gRNAh and 5′-targeting sites are very close to the middle line, the gRNAi site is near the left repeat but away from the right repeat. After CRISPR gene targeting and successful SSA mediated DSB repair, both *MT* and Ld131600 gene are deleted. (i) In WT cells, following gRNAi targeting and Cas9 cleavage, Ku heterodimers immediately bind the DSB ends and hold them together by Ku-Ku interactions, and the cell initiates 5′- to 3′-end resections. With progression of the end resections, Ku translocate inward and continue holding on the two broken chromosomal ends. In gRNAi targeting, because the left single-strand complementary direct repeat sequence is formed much earlier than the right single-strand repeat, Ku binding do not really help anneal these repeats. However, in gRNAh targeting (ii), since the left and the right single-strand repeats are formed at almost the same time and in proximity, Ku binding greatly help anneal these repeats. In the absence of Ku binding in the Ku-deficient cells (iii, iv), the 5′- to 3′-end resections can progress more rapidly to accelerate the DSB repair in the case of gRNAi targeting (iii) compared with in WT cells (i). However, in the case of gRNAh targeting (iv), without Ku binding, the left and the right single-strand repeats are formed at a distance and can move freely so delay the DSB repair compared with in WT cells (ii). See the detailed explanation in the text.

Given that *Leishmania* lacks DNA Ligase IV and several other key NHEJ components, using the Ku mutants generated above, we determined whether *Leishmania* Ku proteins are still involved in DSB repair by binding DNA ends and interfering the 5′- to 3′-end resections. We have previously shown that L. donovani Miltefosine Transport (MT) gene (LdBPK_131590) is an ideal target for studying CRISPR gene targeting and DSB repair efficiency (25, 26, 28). Because the MT located on the cell membrane is responsible for importing Miltefosine (MLF) which is toxic to *Leishmania* parasites, deletion mutations of *MT* gene resulting from CRISPR targeting and successful DSB repair will lead to *Leishmania* resistance to MLF (25, 26, 28). Wild-type (WT), Ku70(-), and Ku70/80(-) L. donovani promastigotes were transfected with a series of CRISPR vectors which express gRNAs targeting various sites of the *MT* gene (Fig. 3B) (26). The MLF resistance rates were determined by placing the transfectants (7-weeks posttransfection) in 96-well tissue culture plates containing MLF.

If the Ku proteins are not involved in DSB repair in *Leishmania*, the Ku null mutants would display similar MLF resistance rates as WT *Leishmania* cells. In contrast, if the Ku null mutants show much higher or lower MLF resistance rates, it would suggest that *Leishmania* Ku proteins are still involved in DSB repair by either an inhibition or promotion mechanism. Interestingly, depending on the gRNA targeting location, *Leishmania* Ku proteins could have opposite effects on DSB repair in *Leishmania*. As shown in Fig. 3A, a total of seven gRNAs were tested. In gRNAi targeted cells, the MLF resistance rates were found to be much higher (~5-fold) in Ku70(-) and Ku70/80(-) mutants than in WT cells, suggesting as expected, Ku70/80 in WT cells are able to bind DSB ends to slow the 5′- to-3′-end resections required for SSA mediated DSB repair. In contrast, a 3- to 7-fold reduction of MLF resistance rates were observed in the Ku null mutants transfected with gRNAh or gRNA5’. Addback of Ku70 in the Ku70(-) mutant restored the MLF resistance rates to the levels observed from WT cells, indicating Ku proteins promote DSB repair in these later circumstances despite obstructing the repair of DSB resulting from gRNAi targeting. However, MLF resistance rates using gRNAe, f, g, and j were similar between WT *Leishmania* cells and Ku null mutants. It is important to note that gRNA activity could vary among gRNAs due to the guard sequence differences, which explains why gRNA e and f had very different MLF resistant rates (26).

Interestingly, further analysis revealed that the targeting sites for those two gRNAs (h and 5′), which displayed much higher MLF resistance rates in WT cells, are near in the middle point between the two direct repeats used for SSA DSB repair (Fig. 3B). In contrast, the targeting site for gRNAi showed a much lower MLF resistance rate in WT cells near the left direct repeat but away from the right repeat. The remaining four gRNA targeting sites are between gRNAi and gRNAh sites (Fig. 3B). Therefore, this suggests that like in other organisms (32–36), *Leishmania* Ku proteins are able to bind the DSB DNA ends, inhibit and slow down the extended 5′- to 3′-end resections required for SSA or MMEJ DSB repair (see diagram Fig. 3B, panel i). However, like the resection-dependent NHEJ pathway recently discovered in mammalian cells (47, 48), with the progress of 5′- to 3′-end resections, the Ku heterodimers could also translocate inward (away from the DNA ends) while keep holding the two 3′-overhangs together through Ku-Ku and other protein interactions (7, 8). In the case of gRNAh and gRNA5’ targeting, the DSBs occurred in the middle between the two direct repeats, thus, the complementary 3′-overhangs would be formed from the two direct repeats around the same time and in close proximity, and thus be easily annealed, so as to speed up the SSA DSB repair process (Fig. 3B, panel ii). However, in the absence of Ku binding, the two 3′-overhangs would be separated and move freely, which could significantly slow down the annealing between the homologous 3′-overhangs although deficiency of Ku proteins could accelerate the 5′- to 3′-end resections (Fig. 3B, panel iv). However, in gRNAi targeting, because the homologous single-strand sequences were formed at a different time and at a distance, even the binding of Ku might hold the two 3′-overhangs together at the single to double-strand DNA transition site, Ku binding in this case would not promote 3′-overhang homologous sequences annealing but delay the 5′- to 3′-end resections (Fig. 3B, panel i and iii). For the remaining four gRNAs, their targeting sites are between gRNAi and gRNAh, and the slight annealing advantage of Ku binding could be repressed by the Ku blocking activity of 5′- to 3′-end resections. As a result, no significant changes in DSB repair efficiency could be observed for the four gRNAs between WT and Ku null mutants. Together, these data indicate that depending on the location of a DSB between two direct repeats, Ku protein binding could have an inhibition effect, no effect, or a promotion effect on the DSB repair by single-strand annealing. Ku proteins accelerate repair if the DSB occurs in the central region between two direct repeats by promoting annealing. In contrast, Ku proteins could impede repair by blocking the 5′- to 3- end resections if the DSB occurs near one direct repeat but away from the other. With the targeting site moving toward the middle, the Ku inhibition effect slowly changes to the promotion effect for SSA mediated DSB repair.

The gRNAi targeting data indicate, as reported in other organisms (32–36), the *Leishmania* Ku70/80 heterodimer could bind and compete for DSB ends against the MRN complex, and block or delay the 5′-end resections. However, the gRNAh and gRNA5’ targeting data in WT- and Ku-deficient cells also suggest that *Leishmania* Ku could translocate inward with progress of 5-′ to 3′-end resections and maintain a hold on the two 3′-single-strand overhangs. Indeed, limited inward Ku translocation was observed in recently discovered resection-dependent NHEJ in mammalian cells (47, 48). Although this Ku translocation model explains well the different DSB repair frequencies detected in WT and Ku-deficient L. donovani cells, human Ku heterodimers bounded on the DNA ends (if the NHEJ cannot be completed) are usually promptly removed by the MRN complex. Human Ku heterodimers can also be removed through Ku70 protein phosphorylation during 5′- to 3′-end resections to initiate the homologous recombination pathway (5, 9, 35, 36). It is surprising that *Leishmania* Ku might be able to translocate inward and hold together the two 3′-overhangs during extensive 5′- to 3′-end resections (more than 4 kb) required for SSA mediated repair in the *MT* gene locus. This could be due to several factors. First, DNA PKcs, DNA Ligase IV, and other NHEJ factors which limit human Ku translocation by forming a synapse between the DNA ends are not present in *Leishmania*. Second, although Ld Ku proteins possess all the main domains of human Ku, they share only 25% amino acid identity. Ld Ku70 contains extra amino acid sequences and is 30 kDa larger than human Ku70, and the phosphorylation sites required for human Ku displacement are not identified in Ld Ku70. The differences in sequence and size may facilitate *Leishmania* Ku translocation and Ku-Ku interactions. Third, the MRN complex required for 5-′ to 3′-end resections may function somewhat differently in *Leishmania* and could be more tolerant to the presence of Ku on the DNA ends. It may, together with other unknown factors or proteins, push L. donovani Ku to slide inward extensively during SSA mediated DSB repair in *Leishmania*. While this translocation model best elucidates the DSB repair rate differences between WT and Ku-deficient cells for gRNAh and gRNA5’ targeting, other mechanisms could also be possible. For example, the 3′-single-strand DNA overhangs may form stem-loop structures, through binding the stem loops, *Leishmania* Ku may hold the two 3′-overhangs together and assist their annealing and repair. Lastly, this Ku translocation model may also suggest it is better to select a gRNA targeting site near the middle between two direct repeat sequences in the genome. Because *Leishmania* mainly depends on SSA to repair DSB, this will likely improve the CRISPR gene deletion efficiency if a selection marker donor is not used.

### L. donovani Ku proteins are dispensable for maintaining the normal lengths of telomeres.

Telomeres are repetitive DNA sequences (commonly TTAGGG repeats) found at the ends of chromosomes that are required to protect chromosome ends, prevent chromosome fusion, and maintain genomic integrity. In mammals, telomeres form a loop (T-loop) together with the six-protein telomere binding shelterin complex to protect chromosomal ends (49). Ku has been shown to directly bind the telomere sequences, interact with the telomere proteins, and help recruit telomerase through associating with the RNA component stem-loop structure. Therefore, Ku is involved in telomere length regulation in variety of organisms. For instance, deletion of the yeast Ku70 or Ku80 gene resulted in decreased telomere length (50, 51). Ku-deficient mice displayed abnormal telomere lengths with telomeric fusions (52). Human cells with a knockdown of Ku80 were reported to have shortened telomeres and increased cell lethality (53). In particular, progressive telomere shortening was observed in Ku-deficient *Trypansoma brucei* (43, 44) which like *Leishmania* also belongs to *Trypanosomatida*. In T. brucei, the telomere sequences ranging from 5 to 14 kb were progressively increased in WT cells but significantly shortened in the Ku80-deficient cells with an increasing or shortening rate of ~100 bp per week (44). In contrast, abnormally long telomeres were observed in the Ku knockout *Drosophila* cells (54). Notably, it has been recently reported that Leishmania mexicana telomeres were elongated from 250 to 850 bp to 250 to 3,000 bp upon deletion of the Ku70 or Ku 80 genes, and the lengths of the elongated telomeres then remain stable (unchanged) in the Ku-deficient L. mexicana cells (45).

**FIG 4:**
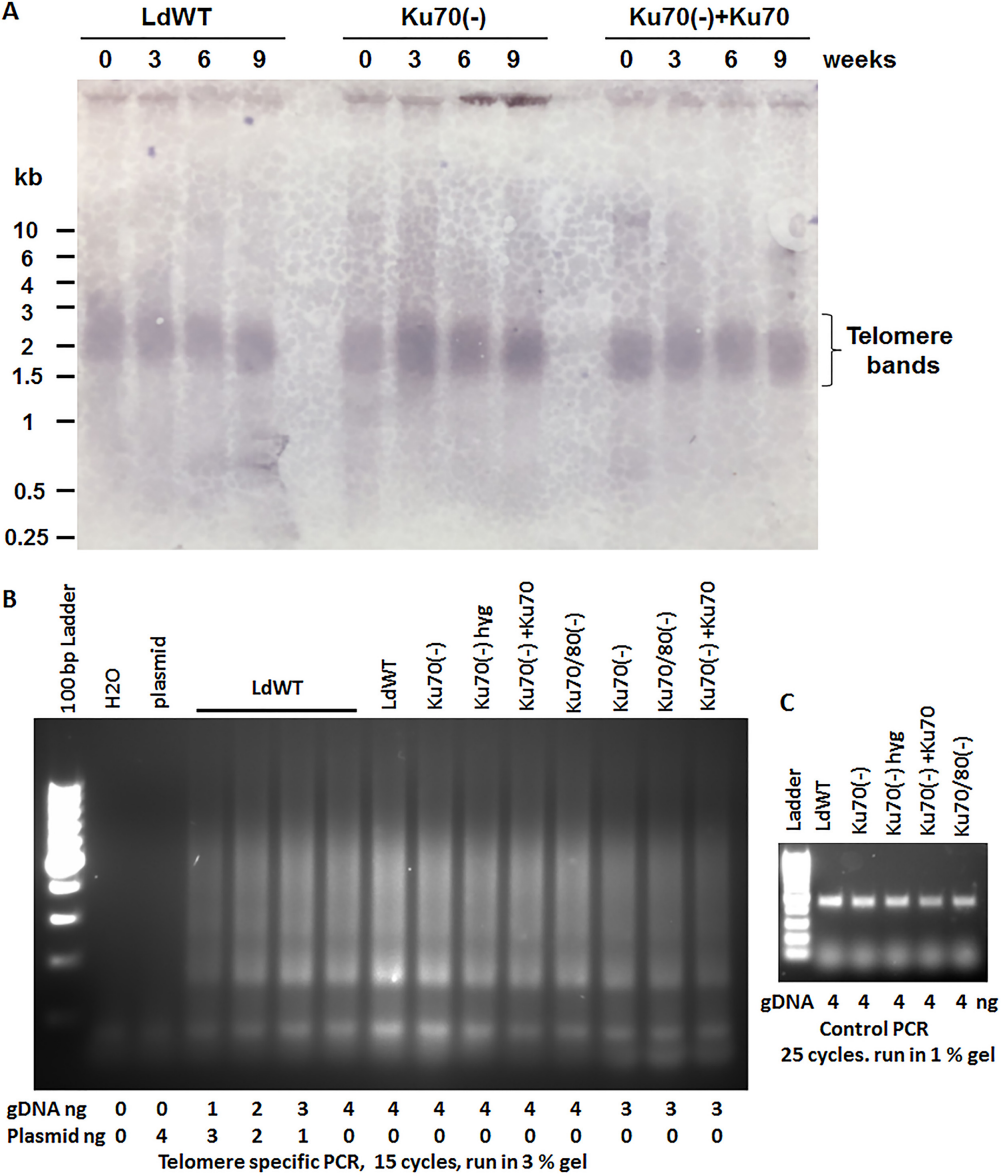
Normal telomere lengths are maintained in Ku-deficient L. donovani. (A) TRF Southern blot analysis. Genomic DNA from wild-type (LdWT), Ku70-deficient cells [Ku70(-)], and from the Ku70 addback cell line [Ku70(-) +Ku70], which expressed an ectopic copy of Ld Ku70 was prepared every 3 weeks for a period of 9 weeks. The DNA (10 μg for each sample) was digested with Msp I and separated by agarose gel electrophoresis, Southern-blotted, and probed with a biotin-labeled telomeric (TTAGGG)_4_ probe. Note, because L. donovani is a diploid organism containing 36 chromosomes, the telomere bands smear detected here represent at least 144 telomeres of different sizes (1.5 to 3 kb). (B) Semiquantitative telomere specific PCR analysis. Equal amounts of genomic DNA(3 or 4 ng) from LdWT, Ku70(-), Ku70(-) cells grown in hygromycin containing medium (Ku70(-)hyg), Ku70(-)+Ku70, and Ku70/80(-) cells were used to set up the telomere specific PCR for a total of 15 cycles and separated in 3% agarose gel. Note, the typical telomere PCR bands (smears) were not detected in the tubes using H2O or plasmid DNA as the PCR template, similar telomere bands (smear) intensities were observed in those L. donovani WT and Ku-deficient cell lines. (C) A semiquantitative PCR to verify that equal amounts of genomic DNA were used in the telomere specific PCR in (B).

To determine whether Ku is also involved in telomere maintenance in L. donovani, we compared the telomeres’ lengths in WT and Ku-deficient L. donovani cells by terminal restriction fragment (TRF) Southern blot and semiquantitative PCR analysis as described in methods (43–45, 55). L. donovani promastigotes were harvested from culture every 3 weeks for total 9 weeks. The genomic DNA prepared from those cell cultures were digested to completion with restriction enzyme Msp I (with CCGG cut site) and subjected to Southern blot analysis with a biotin labeled telomere oligo probe (TTAGGG)_4_. As shown in Fig. 4A, unlike what were reported in T. brucei and L. mexicana (43–45), no progressive telomere elongation or shortening was observed in WT or Ku70-deficient L. donovani cells. All the analyzed L. donovani cells retained similar lengths of telomeres over the 9-week culture period. Depending on the organism, telomere lengths can vary greatly ranging from several hundreds to more than 10,000 bp (56). Large telomere length variation was also observed in different *Leishmania* species and strains; for example, L. mexicana M379 telomeres (250 to 850 bp) are much shorter than the telomeres of L. major LV39 (550 to 13,300 bp) (45, 56). According to the Southern blot shown in Fig. 4A, we estimated the telomere lengths for this L. donovani 1S/Cl2D strain are between 1.5 to 3 kb. In addition, our semiquantitative PCR analysis with telomere specific primers revealed L. donovani Ku70/80-deficient cells also displayed no telomere shortening and contained the similar sizes of telomeres as WT L. donovani cells (Fig. 4B). Taken together, these data indicate L. donovani Ku proteins are dispensable for maintaining the normal telomere lengths.

It is surprising that Ku’s function in telomere regulation could vary so greatly even in genetically closely related *Trypansoma* parasites. Interestingly, amino acid sequence alignments indicate that T. brucei Ku70 and Ku80 proteins are 160 to 220 amino acids smaller than L. donovani Ku proteins and they share only 26% amino acid identities. L. mexicana Ku proteins have similar sizes as L. donovani but they have 9% to 14% differences of amino acids. However, because there are many other genetic background variations between those *Trypanosomatida* parasites, it is not clear whether the differences present in the Ku proteins were directly responsible for the different involvement of Ku proteins in the telomere length maintenance observed among those parasites. Further studies are needed to determine whether L. donovani Ku has any direct or indirect interactions with the telomeres.

### *Leishmania* Ku proteins are required for healthy proliferation of *Leishmania* promastigotes.

**FIG 5:**
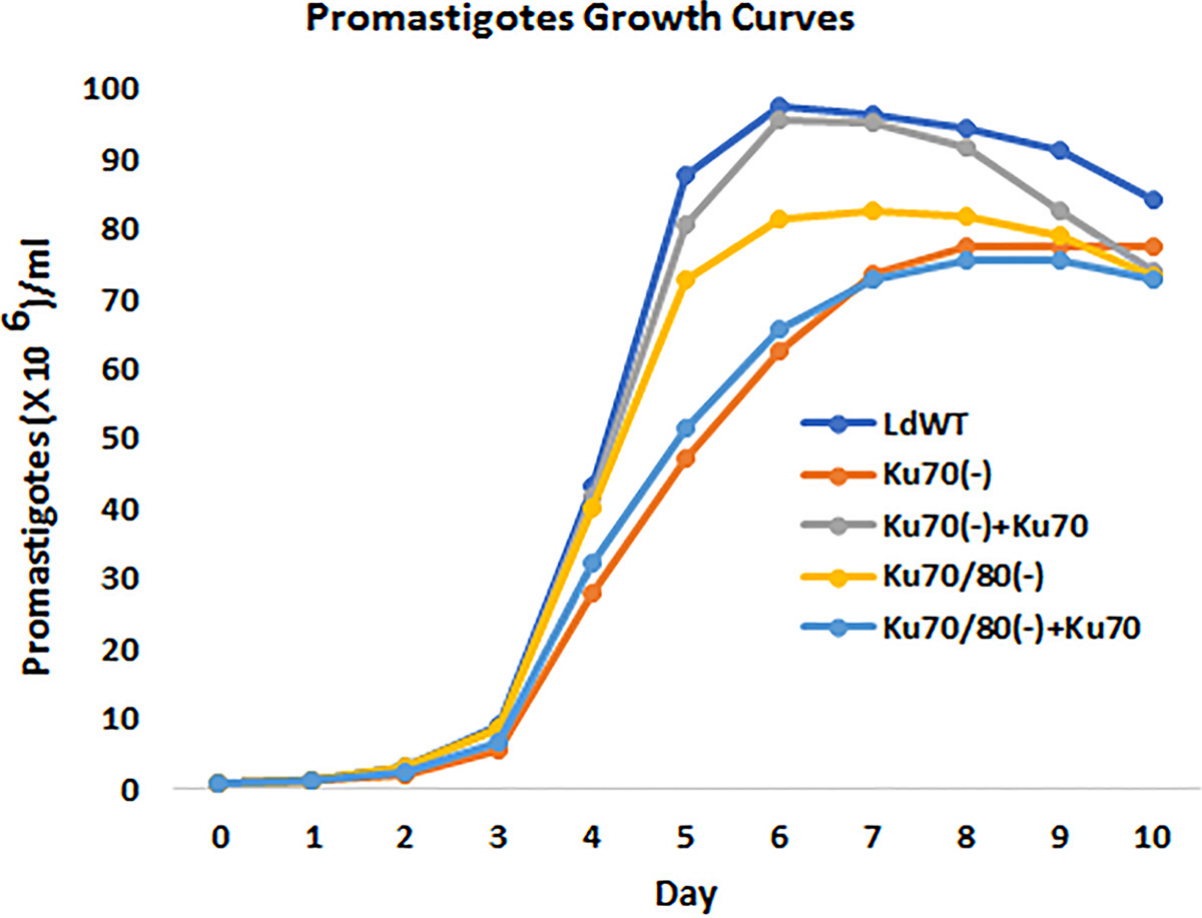
The growth curves of L. donovani WT and Ku-deficient promastigotes. Equal number of L. donovani promastigotes (1 million cells per mL) was inoculated in flask containing 4 mL culture medium. The parasite growth was monitored by microscope counting once a day for total 10 days. This is the representative data of three independent experiments.

We next determined whether Ku deficiency could affect L. donovani promastigote proliferation in culture. As shown in Fig. 5, compared with WT cells, Ku70(-) promastigotes displayed a significant slower growth rate, adding back Ku70 gene was able to restore the growth rate close to the level of WT cells. Surprisingly, disruption of both Ku70 and Ku80 genes had less inhibition effect on cell growth than disrupting Ku70 gene alone. Adding back Ku70 gene in Ku70/80(-) mutant cells was not able to improve the growth rate but further inhibited the cell proliferation as seen in Ku70(-) cells. Because Ku70 and Ku80 normally form a stable heterodimer, Ku80 or Ku70 protein alone is not stable in the cell or may form Ku80 or Ku70 homodimers (7, 8, 57). Taken together, these data indicate that the Ku heterodimer is required to maintain normal growth of *Leishmania* promastigotes, Ku80 or Ku70 protein alone could be toxic to the cell (57).

Besides DSB ends, Ku could also bind other DNA structures and RNA stem loops (7). Ku may interact with other cellular proteins besides the NHEJ factors through the C terminal sequences of Ku70 or Ku80 proteins (7, 8). Aside from NHEJ and telomere maintenance, Ku has been implicated in other cellular processes, including DNA replication, transcription, and translation control (7, 8, 37–39). In this study, we have observed that Ku-deficient L. donovani promastigotes proliferated slower than the WT cells. A similar growth defect was also observed in Ku80 knockout L. mexicana mutants (45), suggesting *Leishmania* Ku could have a broader function in addition to its role in DSB repair. Thus, it will be interesting in future studies to investigate whether *Leishmania* Ku proteins interact with other cellular DNA, RNA structures, and proteins, and how those interactions impact their cellular function.

### Reconstitution of Mycobacterium marinum nonhomologous end joining pathway in *Leishmania*.

Due to the presence of a competent NHEJ pathway, CRISPR gene targeting is very efficient in mammalian cells and in Toxoplasma gondii, making it possible to isolate the CRISPR mutated cell clones directly with no need to use selection marker donors, and to screen the genomes with CRISPR libraries (58–61). In comparison, due to absence of a functional NHEJ pathway, *Leishmania* depends on SSA and MMEJ to repair DSB, which often cause large deletions (see Fig. 3B) and fail (25, 26, 28). The mutation rate of CRISPR gene targeting is often less than 1% in *Leishmania* and antibiotic selection marker donors are typically required to facilitate isolating deletion mutants (25, 28–31).

**FIG 6:**
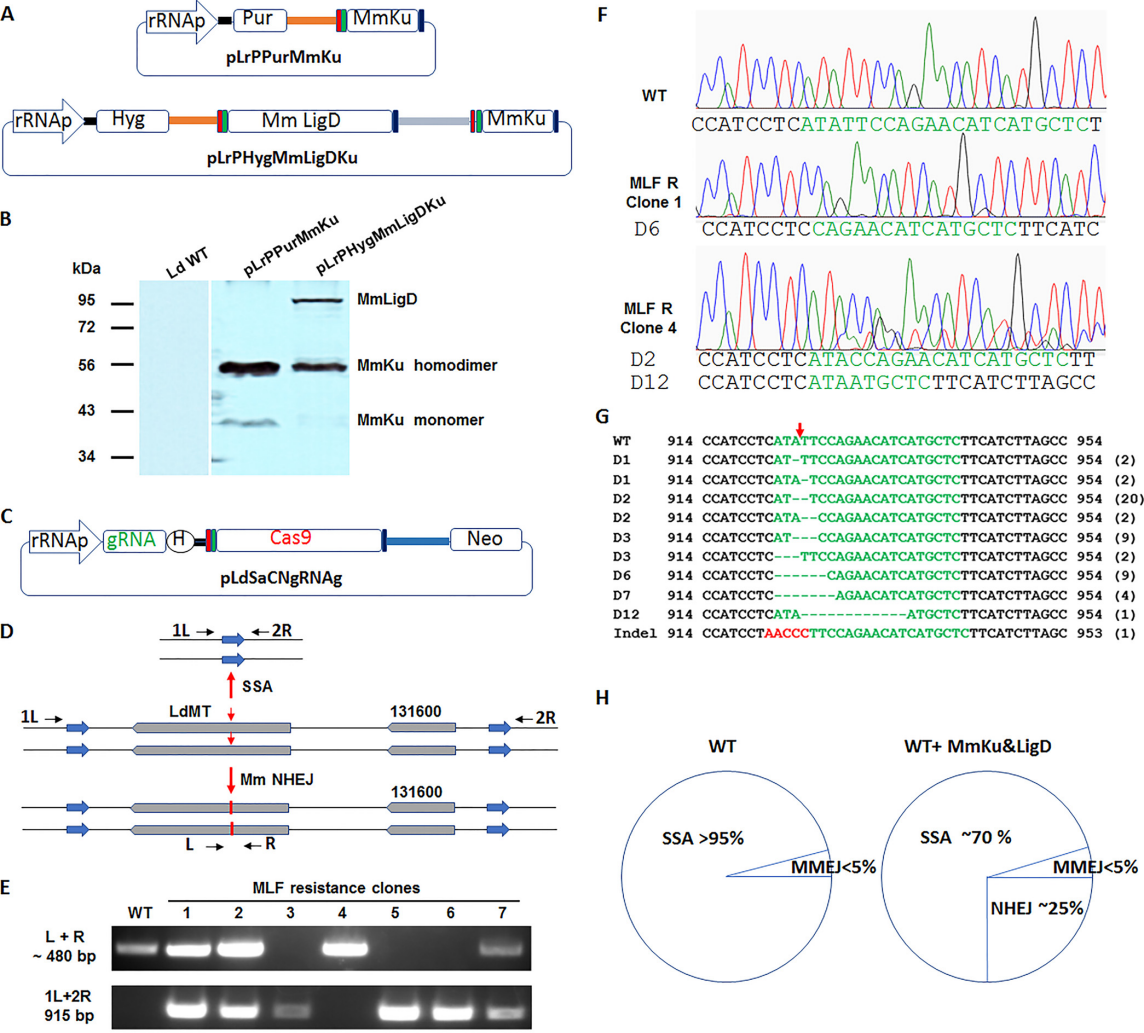
Reconstitution of Mycobacterium marinum NHEJ pathway in *Leishmania*. (A) The plasmids used to express Mm Ku and LigD in *Leishmania*. pLrPPurMmKu conferring puromycin resistance and expressing Mm Ku; pLrPHygMmLigDKu conferring hygromycin resistance and coexpressing Mm LigD and Ku. The rectangle in red, Flag tag; in green, SV40 NLS; in dark blue, Nucleoplasmin NLS. (B) Western blot analysis showing Mm LigD was expressed at the expected size and Mm Ku, however, mainly existed as homodimers in *Leishmania*. (C) The CRISPR vector pLdSaCNgRNAg used to generate DSBs in *MT* gene in the Mm Ku and LigD expressing *Leishmania* cells. (D) SSA and Mm NHEJ were likely the main repair mechanisms following CRISPR targeting of *MT* gene in the Mm Ku and LigD expressing *Leishmania* cells. Note, it is also possible that the DSB in one *MT* gene allele was repaired with Mm NHEJ but in another allele with SSA. The primers used to detect the Mm NHEJ events (L+R) and SSA events (1L + 2R) are also indicated. The expected PCR product is 480 bp for (L+R) primer pair and 9 kb for (1L + 2R) primer pair in the WT locus and 915 bp after SSA. (E) PCR analysis showing that Mm NHEJ repair (~480 bp bands), SSA (915 bp band) or both repair pathways could be detected in the *Leishmania* MLF resistance clones derived from the Mm Ku and LigD expressing cells. (F) Chromatograms showing the gRNAg targeting site (highlighted in green) in WT cells, and the typical small deletions resulted from Mm NHEJ repair in MLF resistance clone 1 and 4. Note, a 2-bp deletion in one *MT* gene allele and a 12-bp deletion in another *MT* gene allele have been detected in clone 4. (G) DNA sequence alignments showing all the Mm NHEJ mediated deletion and insertion events detected in Mm Ku and LigD expressing *Leishmania* cells following CRISPR targeting of *MT* gene with gRNAg. The number of times detected for a deletion or insertion event is indicated in parentheses after each sequence. Note, all the frequently detected NHEJ repairs D2(20), D3(9), D6(9), and D7(4) used 1 or 2 bp microhomology sequences to assist annealing. (H) The frequency of each repair pathway (SSA, MMEJ, and Mm NHEJ) in WT and *Leishmania* cells expressing Mm Ku and LigD. NHEJ could constitute around 25% of total repair events in Mm Ku and LigD expressing cells.

Unlike in eukaryotes, NHEJ pathway in *mycobacteria* is mainly composed of two proteins, Ku and Ligase D. The mycobacterial NHEJ pathway has been successfully reconstituted in yeast and in E. coli by simply introducing the Ku and Ligase D proteins (17–19). To improve DSB repair efficiency and specificity in *Leishmania*, we introduced the M. marinum NHEJ pathway into *Leishmania*. M. marinum Ku and Ligase D were found to be more efficient and accurate in joining DNA ends when expressed in E. coli than M. tuberculosis counterparts (19). M. marinum Ku (Mm Ku) and Ligase D genes were fused with SV40 and Nucleoplasmin nuclear localization signals at their 5′- and 3′-ends for proper nuclear localization and fused with Flag tag for detection in *Leishmania*. Mm Ligase D and Ku genes were ligated into the *Leishmania* expression vector pLrPHyg in tandem to generate plasmid pLrPHygMmLigDKu (Fig. 6A). We also constructed plasmid pLrPPurMmKu with a puromycin selection marker as an additional source to further increase Mm Ku expression in *Leishmania* (Fig. 6A). L. donovani promastigotes were individually or double transfected with these plasmids using hygromycin and puromycin selection. As shown in Fig. 6B, Mm Ligase D was expressed in *Leishmania* at expected 90 kDa size (19). Interestingly, Mm Ku was expressed in *Leishmania* as two bands, the light 38 kDa band is the expected size of a single Mm Ku protein. The dark large band around 56 kDa is most likely the Mm Ku homodimer, a ring shape structure which could migrate faster than linearized proteins (76 kDa), indicating the homodimer was very stable and resistant to separation under current SDS-PAGE running conditions.

To determine whether Mm Ku and Ligase D could form a functional NHEJ pathway in *Leishmania*, WT and *Leishmania* cells expressing Mm Ku and Ligase D were transfected with the *MT* gene targeting CRISPR plasmid pLdSaCNgRNAg (Fig. 6C). Three- to 7-weeks posttransfection, the *Leishmania* cells were then placed in 96-well plates in medium containing MLF to detect the NHEJ mediated DSB repairs, and to determine whether Mm NHEJ improved the overall gene targeting efficiency in *Leishmania*. The genomic DNA extracted from the MLF resistance clones were subjected to PCR and DNA sequencing analysis. WT *Leishmania* cells mainly rely on SSA (>95% of times) and MMEJ (<5% of times) to repair DSBs in *MT* gene locus resulting in 9 kb deletions (26). It is therefore usually impossible to detect PCR bands near 480 bp in MLF resistance cells with primers LR unless the DSBs resulting from gRNAg targeting were repaired using Mm NHEJ pathway instead of SSA (Fig. 6D). As expected, we could not detect the 480 bp LR PCR band in WT *Leishmania* cells transfected with gRNAg CRISPR plasmid after MLF selection (data not shown). However, as shown in Fig. 6E as an example, the ~480 bp PCR products could be detected in more than half (50%) of the MLF resistance clones expressing Mm Ku and Ligase D. Sequencing analysis confirmed that the introduced Mm NHEJ pathway was functional in *Leishmania* and able to repair DSBs and cause the NHEJ distinctive small deletions and insertions (Fig. 6F and G). Interestingly, the most common deletions [D2 (20), D3 (9), D6 (9) and D7(4)] by Mm NHEJ all have used 1 bp (T or C) or 2 bp (TC) microhomology sequences between ends to assist annealing and repair (Fig. 6G). It is also interesting to note that while *MT* gene was mainly inactivated by frame shifts from the NHEJ deletions and insertions, deletion of the single amino acid isoleucine (I308) or phenylalanine (F309) in the MT protein caused by the D3 deletions was responsible for loss of MT activity.

Consistent with the above observation, there was an approximate 2-fold increase of MLF resistance rate in the Mm Ku and Ligase D expressing cells (2.3%) compared with in WT cells (1.2%). Both MT gene alleles are required to be mutated or deleted for the cell to become MLF resistance. We have observed that after CRISPR targeting, both MT gene alleles were mutated by Mm NHEJ (for example clone 4 in Fig. 6E and F) or deleted by SSA (for example, clones 3, 5, and 6 in Fig. 6E), or in combination, that is one allele was repaired and mutated by NHEJ while other allele was completely deleted by SSA mediated repair (for example, clones 1, 2, and 7 in Fig. 6E). As Mm NHEJ repairs were detected in approximately 50% of MLF resistance cells 7-weeks post-CRISPR plasmid transfection and considering that other *MT* gene allele could also be deleted by SSA mediated repair, we estimated that at least 25% of DSBs were repaired by NHEJ in the Mm Ku and Ligase D expressing *Leishmania* cells (Fig. 6H).

**FIG 7:**
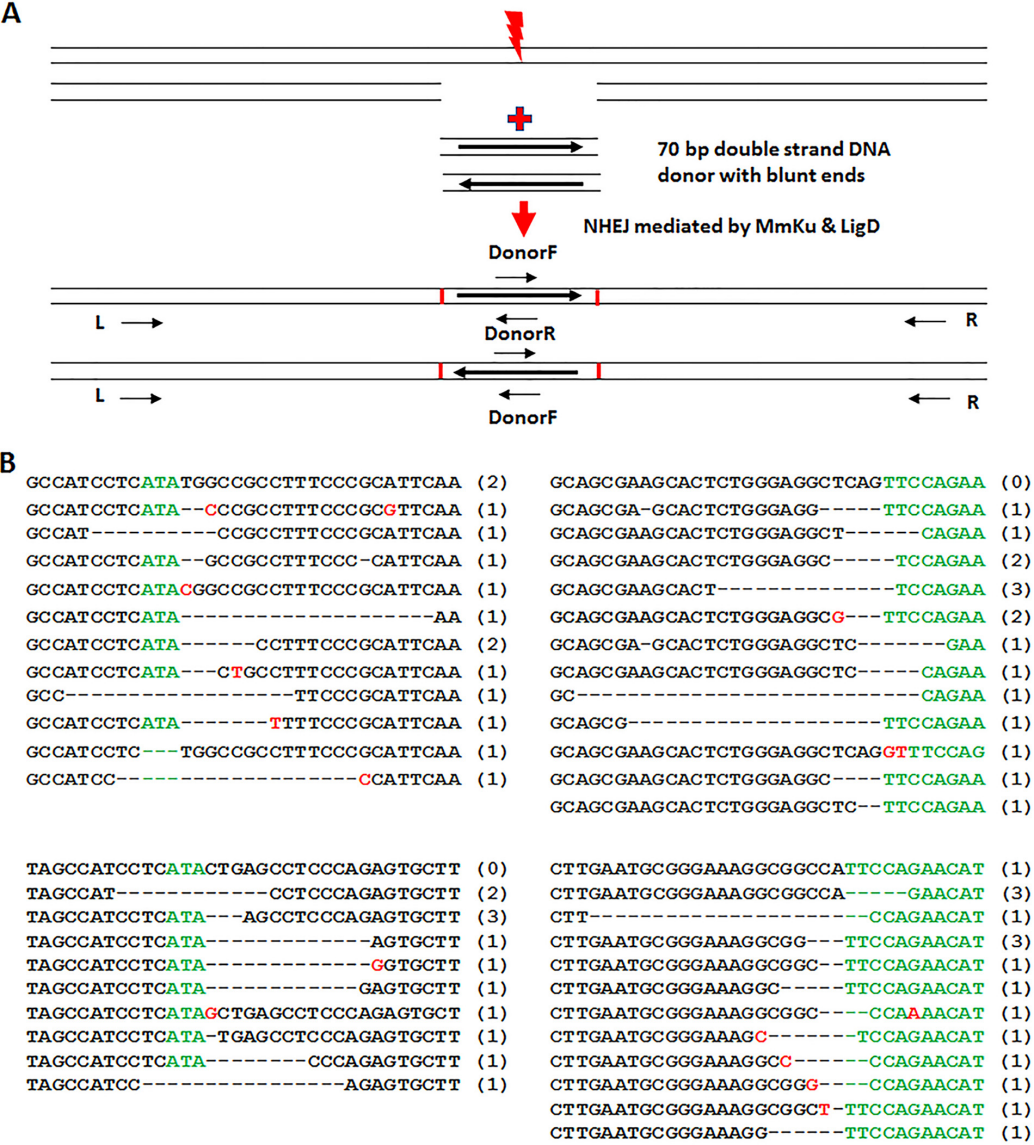
Direct ligation of blunt DNA ends without processing by Mm NHEJ was rarely detected in Mm Ku and LigD expressing *Leishmania* cells. (A) The strategy to create and detect the direct ligation events of blunt DNA ends by Mm NHEJ. (B) DNA sequencing analysis show direct ligation of blunt DNA ends by Mm NHEJ rarely occur in transgenic *Leishmania*. In over 58 clones sequenced, only three direct blunt ends ligation events (5%) were detected; see the top sequences in upper left and lower right panels. Again, except a few small (1 to 2 bp) insertion mutations (in red), most of the remaining clones display various length (1 to 27 bp) of small deletions, many of those deletions occurred between 1 and 2 bp microhomology sequences.

Because CRISPR cleavage by SpCas9 or SaCas9 generates blunt DNA ends, if Mm Ligase D can ligate those blunt DNA ends directly without any ends processing, those faithfully repaired DSBs will not be detected using MLF selection. To determine whether blunt DNA ends could be directly ligated by Mm Ligase D in *Leishmania* cells, a 70-bp double-strand DNA donor with blunt ends were transfected into *Leishmania* cells containing pLhygMmLigDKu and pLdSaCNgRNAg plasmids. Total genomic DNA were prepared 2 days later from those donor transfectants and subjected to PCR with primers (L+ Donor R; Donor F +R; L+ Donor F and Donor R+R) as indicated in Fig. 7A. The four PCR products (~130 bp and 440 bp) were then ligated into pSp72 vector. The individual clones were analyzed by DNA sequencing. Surprisingly, only three direct blunt ends ligations (5%) were detected among total 58 clones sequenced. Except for several small (1 to 2 bp) insertion mutations, most of the remaining clones display various length (1 to 27 bp) of deletions. Although NHEJ, as the name indicates, can join DNA ends without using any homologous sequences, many of those small NHEJ deletions occurred between 1 and 2 nt microhomology sequences (Fig. 6G; Fig. 7B).

The Mm NHEJ DSB repair products with small deletions (1 to 27 bp) detected in *Leishmania* are similar to that observed in yeast expressing M. tuberculosis Ku and Ligase D (17), but different from M. marinum Ku and Ligase D expressing E. coli that were frequently much larger deletions (>1,000 bp) (19). Mm NHEJ in E. coli often used longer microhomology sequence (4 bp) for annealing (19), suggesting factors such as chromosome structure, nucleases, and polymerases from the receipting hosts could also be involved in Mm NHEJ repair.

Although Mm NHEJ mediated repairs can account for a quarter of the total DSB repair events in Mm Ku and Ligase D expressing *Leishmania* cells, further optimization of Mm NHEJ is needed to improve its DSB repair efficiency for CRISPR gene targeting without using a donor DNA in *Leishmania*. Depending on whether a heterologous protein is stable, harmless, or toxic, its expression level could vary greatly with time in *Leishmania* (62). It appears the proper expression level and ratio of Mm Ku and Ligase D are necessary for making efficient Mm NHEJ repairs since overexpression of Mm Ku alone may inhibit DSB repair (20, 34, 35, 63). Indeed, we have observed the total DSB repair rate could sometimes be lower in the Mm Ku and Ligase D expressing *Leishmania* cells (along with low NHEJ repair frequency) than in WT cells (data not shown). This could be due to several reasons. First, imbalanced overexpression of Mm Ku could lead to multiple Ku homodimers binding on DSB ends which may prevent proper ends processing and ligation by Ligase D (20, 63). Second, even after successful sealing of the break by Ligase D, *Leishmania* may have difficulty removing the extra trapped Ku homodimers on the DNA which may prevent DNA replication resulting in cell death because *Leishmania* may not have the specific and efficient mechanisms to remove Mm Ku promptly after end joining (36, 63, 64). Lastly, if an Mm NHEJ cannot be completed because of a low expression level of Ligase D, like Ku heterodimer in mammalian cells and Ld Ku shown in this study, Mm Ku binding could prevent 5′-end resections and inhibit DSB repairs by SSA and MMEJ (32–36). Moreover, we also observed that the DSB repair rates were 2 to 4 times lower in WT *Leishmania* cells than in LdKu70/80-deficient cells, when both cell lines were transfected with the Mm Ku and Ligase D expressing plasmids and targeted with *MT* targeting gRNAg or gRNAj. The Mm NHEJ-mediated repairs were rarely detected in those WT and LdKu70/80-deficient cells (data not shown). These may suggest that the balanced expression of Mm Ku and Ligase D was not achieved in those WT and LdKu70/80-deficient cells, and Mm Ku might assist Ld Ku to block 5′- to 3′-end resections. Further studies are therefore needed to find a way, for example by integrating Mm Ku and Ligase D genes into *Leishmania* chromosomes, which can express Mm Ku and Ligase D stably and in a proper ratio in *Leishmania*. Thus, before the current Mm NHEJ system in *Leishmania* can be optimized to improve efficiency, the most effective and specific CRISPR gene targeting method for *Leishmania* still requires the use of an oligo or selectable marker donors as demonstrated by disrupting the Ku70 and Ku80 genes in this study (25, 28–31).

Owing to the presence of a competent NHEJ pathway, CRISPR gene targeting is very efficient in Toxoplasma gondii with more than 90% gene mutation rate, making it possible to screen the genome with CRISPR libraries (61). In comparison, without using a donor, the mutation rate of CRISPR gene targeting in *Leishmania* is often less than 1% (25, 26, 28). Like *Leishmania*, NHEJ is not present in many eukaryotic parasites, including *Entamoeba*, *Cryptosporidium*, *Theileria*, *Plasmodium*, Giardia, *Trichomonas*, and *Trypanosoma* (65). Although further optimization is required, this study provides an example that the mycobacterial two components NHEJ pathway can be reconstituted in an eukaryotic parasite. It is possible that this M. marinum NHEJ may function more efficiently in a different parasite, which could be used to improve the CRISPR gene targeting (without using donors) specificity and efficiency in those NHEJ lacking parasites.

## MATERIALS AND METHODS

### *Leishmania* strains and culture medium.

L. donovani 1S/Cl2D promastigotes were cultured at 27°C in M199 medium (pH 7.4) supplemented with 10% heat-inactivated fetal bovine serum, 40 mM HEPES (pH 7.4), 0.1 mM adenine, 5 mg l^−1^hemin, 1 mg l^−1^ biotin, 1 mg l^−1^ biopterine, 50 U mL^−1^ penicillin, and 50 μg mL^−1^ streptomycin. *Leishmania* promastigotes were passaged to fresh medium at a 40-fold dilution once a week.

### gRNA design and cloning.

The gRNAs were designed manually or with the aid of Eukaryotic Pathogen CRISPR guide RNA Design Tool (EuPaGDT) (http://grna.ctegd.uga.edu/) to identify off target sites in the genome and the microhomology sequences flanking the DSB. A single gRNA guide coding sequence was ordered as standard oligos with 5′-TTGT and 5′-AAAC overhangs. All oligos, primers, and oligo nucleotides donors used in this study were ordered from Alphaadn (http://alphaadn.com/). The optimal guide length is 19 or 20 nt for SpCas9 gRNA and 21 nt for SaCas9 gRNA. After phosphorylation and annealing, the gRNA guide coding sequences were ligated into the Bbs I site of pLdCN or pLdSaCN CRISPR vector as described (46).

The oligonucleotide sequences used for targeting Ld Ku70 gene (LdBPK_291140):

Ld291140 + 5′-TTGTCCCAGAGTGCTTCGCTGAAC

Ld291140- 5′-AAACGTTCAGCGAAGCACTCTGGG

Ld291140donor 5’TGGCCGCCTTTCCCGCATTCAAGTTCAGTGAGTGAATTCTAGCGGCAGCGAAGCACTCTGGGAGGCTCAG

Ld291140F 5′-TCGTGGCGATTGTGTTGTAC

Ld291140R 5′-TGGTTCGAAACGGCAAAGAG

Donorp 5′-CAGTGAGTGAATTCTAGCGG

The oligonucleotide sequences used for targeting Ld Ku80 gene (LdBPK_300340):

Ld300340 + 5′-TTGTCGGATTCACAGGGGAAGTTCA

Ld300340- 5′-AAACTGAACTTCCCCTGTGAATCCG

Ld300340donor:

5′-GCACCCACGGATTCACAGGGGAAGTCAGTGAGTGAATTCTAGCGGTCAACGAATTCACCGCAGGTGAAGC

Ld300340F 5′-ACTTCATCGACGCCCTTCA

Ld300340R 5′-GAGAGTGAACAGCTGCGTG

Donorp 5′-CAGTGAGTGAATTCTAGCGG

### Expression vector construction.

NEBuilder HiFi DNA Assembly Master Mix from New England Biolabs was used to make all the expression constructs in this study. NEBuilder DNA Assembly Mix allows for directional and seamless assembly of DNA fragments with overlaps (15 to 30 bp), regardless of fragment length or end compatibility. The double-stranded fully sealed DNA vectors can be used for direct transformation of E coli. To facilitate assembly and ligation with NEBuilder DNA Assembly Mix, DNA fragments, including genes and intergenic sequences, were prepared using PCR primers containing additional 20- to 30-bp overlap sequences (see below underlined sequences in primers) with the vectors. The DNA assembly reactions were set up following the manufacture’s instruction. Briefly, the vector and insert sequences were purified from the agarose gel and mixed at 1:2 molar ratio (vector: insert). Five μL of the vector and insert sequences mix was then mixed with equal volume (5 μL) NEBuilder HiFi DNA Assembly Master Mix (total 10 μL). The assembly reaction was incubated at 50°C for 15 min; 5 μL of the assembly reaction was used for transformation of E coli. The correct clones were verified by size, restriction enzyme digestion, and DNA sequencing.

The *Leishmania* expression vector pLhyg was constructed as follows: (i) The 5′-intergenic sequence for LdCL351320 gene was amplified from L. donovani genomic DNA with primers LdCL3513205’F (5′-AGCACTCGTCCGGGATCATCATGATGCACGCAGCGCGTCG) and LdCL3513205’R (5’-GACTCACTATAGGGAGACCGGCAGATCTGATATCTTTGCTTGGTGGATGGAG). LdCL3513205’R primer also contains an EcoR V site (GATATC) to facilitate subsequent cloning. (ii) The PCR fragment (~700 bp) from step (i) was directly ligated with NEBuilder DNA Assembly Mix into Bgl II linearized pSPhyg vector downstream to the hygromycin resistance gene to generate a novel *Leishmania* expression vector pLhyg.

L. donovani Ku70 expression vector (pLhygLdKu70) was constructed as follows: (i) L. donovani Ku70 gene was PCR amplified from L. donovani genomic DNA with primer Ld291140F2 (5’-CTCCATCCACCAAGCAAAGATCAGCTCACCATGGATGAATTTG) and Ld291140R2 (5’-TAGGGAGACCGGCAGATCTGATATCAGGATGGCAGCGGAAAATGCTG) to get the 2950 bp gene fragment. (ii) The PCR fragment from step (i) was directly ligated into the EcoR V linearized pLhyg vector to generate pLhygLdKu70.

Mycobacterium marinum DNA Ligase D expression vector (pLhygMmLigD) was generated by overlap PCR and NEBuilder HiFi DNA Assembly as follows: (i) Three individual PCR fragments were first obtained. Primers MmLigDF1 (5’-CTCCATCCACCAAGCAAAGATGGACTATAAGGACCACGAC) and MmLigDR1 (5’-CAGGCCTGCGCCGAACCCATACCGACCTTCCGCTTCTT) with pLdCN vector as the template were used to get the ~150-bp sequence coding for 3X Flag and SV40 nuclear localization signal (NLS); Primers MmLigDF2 (5’-GAAGGTCGGTATGGGTTCGGCGCAGGCCTGG) and MmLigDR2 (5′-TCGTGGCCGCCGGCCTTTTTTCGCGCACCACCTCACTC) with pDUBmLig vector (19) as the template were used to get the 2,313 bp MmLigD gene fragment; Primers MmLigDF3 (5′-GAGTGAGGTGGTGCGCGAAAAAAGGCCGGCGGCCACGA) and MmLigDR3 (5′-TAGGGAGACCGGCAGATCTGATATCACTTTTTCTTTTTTGCCTGGC) with pLdCN as the template were used to get the ~90-bp sequence coding for nucleoplasmin NLS. (ii) The above three PCR products were mixed and used as the PCR templates with primers MmLigDF1 and MmLigDR3 to get the ~2,500-bp overlap fragment encoding MmLigD fused with Flag tag and two NLS sequences. (iii) The ~2,500-bp fragment from step (ii) was directly ligated into the EcoR V linearized pLhyg vector to generate pLhygMmLigD.

Mycobacterium marinum Ku expression vector (pLhygMmKu) was also generated by overlap PCR and NEBuilder HiFi DNA Assembly as follows: (i) Three individual PCR fragments were first obtained. Primers MmLigDF1 (5’-CTCCATCCACCAAGCAAAGATGGACTATAAGGACCACGAC) and MmKuR1 (5’-CCTTCCAGATGGAGCGCATACCGACCTTCCGCTTCTT) with pLdCN vector as the template were used to get the ~150-bp sequence coding for 3X Flag and SV40 nuclear localization signal (NLS); Primers MmKuF2 (5’-GAAGGTCGGTATGCGCTCCATCTGGAAGGGT) and MmKuR2 (5′-TCGTGGCCGCCGGCCTTTTGGACTTGGCAGCTTGTTTGG) with pDUBmKu vector (19) as the template were used to get the 876-bp MmKu gene fragment; Primers MmKuF3 (5′-CCAAACAAGCTGCCAAGTCCAAAAGGCCGGCGGCCACGA) and MmLigDR3 (5′-TAGGGAGACCGGCAGATCTGATATCACTTTTTCTTTTTTGCCTGGC) with pLdCN as the template were used to get the ~90-bp sequence coding for nucleoplasmin NLS. (ii) The above three PCR products were mixed and used as the PCR templates with primers MmLigDF1 and MmLigDR3 to get the ~1,000-bp overlap fragment encoding MmKu fused with Flag tag and two NLS sequences. (iii) The ~1,000-bp fragment from step (ii) was directly ligated into the EcoR V linearized pLhyg vector to generate pLhygMmKu.

Mycobacterium marinum DNA ligase D and Ku coexpression vector (pLhygMmLigDKu) was generated as follows: (i) The ~1,000-bp α tubulin intergenic sequence was amplified from L. donovani genomic DNA with primers TublinF2 (5′-CCAGGCAAAAAAGAAAAAGTGATGGTACACTCGTGCCGCGCGCTGA) and TublinR1 (5′-TAGGGAGACCGGCAGATCTGATATCCATGGCTGAAAAAGAAGAAAGAG). (ii) The 1,000-bp α tubulin intergenic sequence was directly ligated into the EcoR V linearized pLhygMmLigD vector downstream of LigD to generate the intermediate vector pLhygMmLigD-tubulin. (iii) Primers MmKuF1 (5′-CTCTTTCTTCTTTTTCAGCCATGGATTATAAGGACCACG) and MmLigDR3 with plasmid pLhygMmKu as the template were used to get the ~1,000-bp fragment encoding MmKu fused with Flag tag and two NLS sequences. (iv) The 1,000-bp Ku fragment from step (iii) was directly ligated into the EcoR V linearized pLhygMmLigD-tubulin vector to generate the coexpression vector pLhygMmLigDKu.

To improve expression of MmLigD and Ku in *Leishmania*, the L. donovani ribosome RNA promoter sequence was inserted upstream of the hygromycin resistance gene in pLhygMmLigDKu vector: (i) Primers LdrRNApF3 (5′-CTGCAGGTCGACTCTAGAGGATCCATTGCTTCGCGCCCCAGTACTGGAG) and LdrRNApR5 (5′-GAGAGAGCTCGGTACCCGGGGAGCGCGCTGCCACGTACAGTTG) with L. donovani genomic DNA as PCR template were used to get the ~340-bp L. donovani ribosome RNA promoter fragment. (ii) The ~340-bp ribosome RNA promoter fragment was directly ligated into the BamH I linearized pLhygMmLigDKu plasmid to generate pLrPhygMmLigDKu.

Mycobacterium marinum Ku expression vector (pLrPPurMmKu) with L. donovani ribosome RNA promoter and Puromycin resistance gene was generated as follows: (i) The ~450-bp DNA fragment containing L. donovani ribosome RNA promoter and a 96-bp pyrimidine track was derived from pLrPhygMmLigDKu with primers LdrRNApF3 (5′-CTGCAGGTCGACTCTAGAGGATCCATTGCTTC GCGCCCCAGTACTGGAG) and PurR1(5′-GTACTCGGTCATATCAATTCGAGCTCTGGAG). (ii) The 600-bp Puromycin resistance gene was derived from pGL58 plasmid with primers PurF1 (5′-GCTCGAATTGATATGACCGAGTACAAGCCCAC) and PurR2 (5′-GGAGCATATACGCCCGGAGCCGTCA GGCACCGGGCTTGCGGGTC). (iii) The fragments from step (i) and (ii) were joined by overlap PCR with primers LdrRNApF3 and PurR2. (iv) pLhygMmKu was digested with BamH I and Sac II to remove the hygromycin resistance gene. (v) The fragments from step (iii) and (iv) was joined by NEBuilder HiFi DNA Assembly mix to generate plasmid pLrPPurMmKu.

### Parasite transfection.

Next, 2 × 10^7^ Leishmania donovani promastigotes (middle log phase to early stationary phase) were harvested and washed once in 200 μL Tb-BSF buffer (90 mM Na_2_HPO_4_, 5 mM KCl, 0.15 mM CaCl_2_, 50 mM HEPES, pH 7.3), and resuspended in 100 μL Tb-BSF buffer in a 2-mm gap electroporation cuvette. Then, 2 to 5 μg CRISPR or other vectors in a volume < 20 μL were added and mixed. The transfection was performed with the LONZA Nucleofector 2b Device (program U33). The transfected *Leishmania* cells were selected with 50 μg/mL G418,100 μg/mL hygromycin, or 30 μg/mL puromycin the following day. Once the CRISPR vector transfected cell culture was established, those cells could be then transfected with the donors. To improve the targeting efficiency, 8 μL 100 μM oligonucleotide donor was used for each transfection every 2 days for total of 3 times before cloning the transfectants in 96-well plates. To reconstitute Mycobacterium marinum NHEJ pathway in *Leishmania*, the MmLigD and Ku coexpression plasmid pLrPhygMmLigDKu was first transfected into *Leishmania* cells with hygromycin selection, followed by transfections with plasmid pLrPPurMmKu using puromycin selection and a series of CRISPR vectors targeting the *MT* gene using G418 selection.

### Determine MLF resistance rate and clone MLF resistant cells.

For *Leishmania* promastigotes transfected with various CRISPR vectors targeting *MT* gene, the MLF resistance rate was determined by limiting dilution culture in 96-well tissue culture plates containing 100 μL medium with 40 μM MLF. Depending on estimation of the MLF resistance rate, 2,000 to 8 million *Leishmania* promastigotes (100 μL per well) were inoculated into the first column of 96-well plates in quadruplicate for each transfected cell line, the cells in the first column were then serially 2-fold diluted until the last column in the plate. The plates were sealed and incubated in a 27°C incubator for 2 to 3 weeks. The MLF resistance rates (or the diallelic *MT* gene mutation frequency) were calculated by identifying the furthest MLF surviving wells. The surviving *Leishmania* cell clones in the farthest wells after MLF selection were directly subjected to genomic DNA extraction with M-Fast PCR Genotyping kit (ZmTech Scientifique, Montreal) or expanded in 24-well plates for subsequent genomic DNA extraction and PCR analysis.

### Genomic DNA preparation and PCR analysis.

Parasite genomic DNA were extracted from WT *Leishmania* promastigotes and various MLF resistant cells with the minipreparation method (66) which includes phenol-chloroform extraction and ethanol precipitation. The purity and quantity of those genomic DNA were assessed by Nanodrop spectrophotometer. The parasite genomic DNA were also prepared with M-Fast PCR Genotyping kit (ZmTech Scientifique, Montreal). Briefly, 100 μL stationary phase *Leishmania* culture was harvested by centrifugation at 1,500 g for 5 min, and the cell pellet was resuspended in 15 μL reagent-A and incubated at 95°C for 30 min in a PCR apparatus. Once the tube cooled to room temperature, 3 μL reagent-B was added into the parasite tube and mixed well. Then, 1 μL lysate supernatant was added into 11 μL PCR mastermix for a total 12 μL reaction.

Primers were designed manually or using Primer3 (http://bioinfo.ut.ee/primer3-0.4.0/). Optimal primer length was 20 nucleotides with 60°C Tm. The primers used to detect the NHEJ and SSA specific bands in Fig. 6D and E are as follows: L, 5′-GCTGTTCCGAAACTTGAAGC; R, 5′-GTCGATCGGATTACCGAGAG. 1L, 5′-ACCTCCACCTAAACGGCTCT; 2R, 5′-TCGATCTCGTAATGCGTGAG. The various *Taq* DNA polymerases used in this study include all in one 2X Green PCR Mastermix (ZmTech Scientific, Montreal, Canada), 2X DreamTaq Green PCR Master MIX, and 2X Platinum SuperFi PCR Master Mix (Thermo Fisher Scientific). The PCR program was set up according to manufacturer’s instruction with variation in annealing temperature, extension time, and PCR cycles. The PCR products were separated in 1% to 1.5% agarose gel. The putative NHEJ, MMEJ, and SSA specific bands were extracted from the gel and sent to Genome Quebec Sequencing Center for sequencing confirmation.

### Terminal restriction fragment Southern blot analysis.

The genomic DNA were extracted from WT, LdKu70(-), and Ku70 addback cell lines at 0, 3, 6, and 9 weeks of culture. The same amount of genomic DNA (10 μg) was digested to completion in 300 μL reaction with 50 units restriction enzyme Msp I at 37°C overnight. The Msp I digested genomic DNA were then ethanol precipitated and separated on 1% agarose gel. After denaturing in 1.5 M NaCl 0.5 N NaOH solution for 1 h and neutralization in 1.5 NaCl 1 M TRIS HCl for 30 min, the DNA were transferred to a nylon membrane with 20 × SSC (3M NaCl and 0.3 M Na_3_Citrate) and a stack of paper towels overnight. The membrane was UV-cross-linked for 5 min and baked at 80°C for 30 min. The membrane was prehybridized at 42°C for 1 h in 35 mL prehybridization solution consisting of 1% SDS, 1 M NaCl, and 10% dextran sulfate. The 24 nucleotides telomere oligo probe (5′ Biotin-TTAGGGTTAGGGTTAGGGTTAGGG-Biotin 3′) labeled with biotin at both 5′- and 3′-ends (http://alphaadn.com/) (50 nM) was then added into the prehybridization solution and incubated at 42°C for 2 h. The membrane was washed twice with 2 × SSC, 0.1% SDS for 10 min each at room temperature, and with 0.1 × SSC, 0.1% SDS once for 20 min at 65°C. The biotin-labeled DNA probe hybridized to the target DNA on the membrane was detected with alkaline phosphatase-conjugated streptavidin and the substrate, BCIP-T (5-bromo-4-chloro-3-indolylphosphate, p-toluidine salt) using the Biotin Chromogenic Detection Kit (Thermo Scientific).

### Semiquantitative telomere specific PCR analysis.

The telomere specific PCR used for estimating telomere length was performed as described (50) with telomere specific primers: tel 1: 5′-GGTTTTTGAGGGTGAGGGTGAGGGTGAGGGTGAGGGT; and tel 2: 5′-TCCCGACTATCCCTATCCCTATCCCTATCCCTATCCCTA. The PCR primers (Ld323480L, 5′-AGCTCCTGTGCACGCTTATT and Ld323480R, 5′-GCTGAATCGTCTCCTCGTTC) derived from a single copy gene LdBPK_323480 were used in a semiquantitative PCR to estimate that equal amounts of genomic DNA were used in those telomere specific PCR.

### Western blot analysis.

*Leishmania* promastigotes were harvested by centrifuge at 1,300 g for 5 min and washed once with PBS. Parasites pellets were then resuspended in 1 × SDS sample loading buffer (22.5 mM Tris HCl pH 6.8, 5% Glycerol, 1% SDS, 2.5% β-mercaptoethanol, and 0.025% Bromphenol blue) to make the lysate concentration about 5 × 10^7^ *Leishmania* cells per 20 μL, boiled in water bath for 3 min, and centrifuged at high speed for 10 min. The cell lysate supernatant (equals 5 × 10^7^ promastigotes in 20 μL per lane) was separated in 10% sodium dodecyl sulfate-polyacrylamide gel and transferred to 0.45 μM nitrocellulose membranes (GE) using Bio Rad semidry transfer cell. The membrane was blocked with 10 mL 10% skim dry milk in PBS-T buffer (0.1% Tween 20 in PBS) at room temperature for 1 h, then incubated with the primary anti-flag M2 antibody (Sigma-Aldrich) in 5% skim dry milk-PBS-T solution at 1:5,000 dilution for 2 h. After washing 3 times with PBS-T, the membrane was incubated with the secondary anti-mouse NA931V antibody (GE Healthcare), anti-mouse IgG HRP in 1:5000 dilution in 5% skim dry milk PBS-T solution for 1 h. Detection was performed with Spray-on ECL reagent (Cat# E208085 from ZmTech Scientifique, Montreal) according to manufacturer’s instructions.

### Growth curves.

*Leishmania* promastigotes were seeded at the density of 1 × 10^6^ promastigotes/mL in 4 mL M199 media in 25 cm^2^ flasks and counted by a microscope daily for total 10 days. A minimum of three biological replicates were evaluated.
