# Supplementary material for: Reconstitution of Mycobacterium marinum Nonhomologous DNA End Joining Pathway in Leishmania
Source: mSphere. 2022 Jun 13;7(3):e00156-22. doi: 10.1128/msphere.00156-22 (PMC9241504; doi:10.1128/msphere.00156-22)
Supplement: FIG S1 [file msphere.00156-22-s0001.docx]

1. **Amino acids alignment of human Ku70 and *Leishmania donovani* Ku70**

** Hu Ku70 1 -------------MSGWESYYKTEG---DEEAEEEQEENLEASGDYKYSGRDSLIFLVDASKAMF-------------ES 51**

** Ld Ku70 1 MDEFAEWGEGTTGVVAADSTY-TEGLDLDEFREQLWQRNQ----------RDAVVCLVDCNEGMFGVLPAGESAKTTQTG 69**

** Hu Ku70 52 QSED-------------------------------------ELTPFDMSIQCIQSVYISKIISSDRDLLAVVFYGTEKDK 94**

** Ld Ku70 70 KNGEGAPAHTRGVTKLALLTIGQGAAMSSGAAGGSKAAVGSSPSFFSMTMQCILALLKEKMMCGSKDVVAIVLYNTRTSA 149**

** Hu Ku70 95 NSVNFKNIYVLQE---LDNPGAKRILELDQF--KGQQGQKRFQDMMGHGSD----------------------------- 140**

** Ld Ku70 150 PSTGFRGVYVMQEATRIGTECMQKVEQLEAAGAPGSVAYEEFEARIGHWPTASTSPALAAASSASGRAEPKAALPAALAA 229**

** Hu Ku70 141 ---YSLSEVLWVCANLFSDVQF--KMSHKRIMLFTNEDNPHGNDSAKASRARTKAGDLRDTGIFLDLMHLKKPG------ 209**

** Ld Ku70 230 FPAFKFSEALWEAQRILLSLRSVQAIRHRRLFVFTNRDDPSGGDAHEWNLCRSRACDLGKEGVVLEVFGFGNAGSGGGPH 309**

** Hu Ku70 --------------------------------------------------------------------------------**

** Ld Ku70 310 TSGISKVAGTVANTSRGELPISLTAPVGADGTGSCSSGSSASPLFAVSNQRGQSLDPAAATTDTTIGQSGSGQSDSPSPA 389**

** Hu Ku70 210 --GFDISLFYRDII-----------SIAEDEDLRVHFEESSK----------------------LEDLLRKVRAKETRKR 254**

** Ld Ku70 390 ATGFVQDFFWGPLLREMQMAAARFRASGDGDDLVALAEARGEAFVSGGEGAIYMNSGAGALQQLLVSVVRRAHPQRPFRH 469**

** Hu Ku70 255 ALSRL------------------------KLKLNKDIVISVGIYNLVQKALKPPPIKLYRETN----------------- 293**

** Ld Ku70 470 CLLRIGGLSGTSTALSATTAAATARDEEEALRVAAVPRMAVSLYVPLMRARLPQREWLDGRTNRMLRRVVHLNARTTAAG 549**

** Hu Ku70 294 ----------------------EPVKTKTRTFNTST--GGLLLPSDTKRSQIYGSRQIILEKEETEELKRF----DDPGL 345**

** Ld Ku70 550 RDSDDAGAGNKEGDSGSPTKQLQRLRNEKNCTSESMVRQEDVDPDDLCYYAPVGKERVYFTSEERKRMVEVAATGTEPGF 629**

** Hu Ku70 346 MLMGFKPLV-LLKKHHYLRPSLFVYPE-ESLVIGSSTLFSALLIKCLEKEVAALCRYTPRRNIPPYFVALVPQEE----- 418**

** Ld Ku70 630 TVLLFKDLVDAVKREHVVRRSSFLHSCVQRGGAHSHRLFVLFVRRLRAKQKVAIAQYCSSTTTAPRLVALVPSPDLTAHP 709**

** Hu Ku70 419 ELDDQKIQVTPPGFQLVFLPFADDKRKMPFTEKIM--------------ATPEQVGKMKAIVEKLRFTYRSDSFENPVLQ 484**

** Ld Ku70 710 EKRDQ-VPVDGMGLYVVPLPYAEELRAVPELRTCTRVSKHATPVLADSSVDPTHLELAKQVVSALTVSYQVDAVLNPALQ 788**

** Hu Ku70 485 QHFRNLEALALDLM-----------------------------EPEQAVDLTLPKVEAMNKRLGSLVDEFKELVYPPDYN 535**

** Ld Ku70 789 RQYRKLQELARQFFPLADNPLHPAGGTALAKAGEEGPSTDGAEKTPQELDNTLPDYEGM-QRFAALFQSFNKEVLGKDYN 867**

** Hu Ku70 536 -----PE----GKVTKRKHDN-------EGSGSKRPKVEYS------EEELKTHISKGTLGKFTVPMLKEACRAYGLKSG 593**

** Ld Ku70 868 AFLYCPQPRVAGSATRRPRDSAAGAGAMEAAGPTAASAEEDACNVSIEELIRRAAAENAWDGLIIPQLKEYLATANVSSG 947**

** Hu Ku70 594 L--KKQELLEALTKHFQD-- 609**

** Ld Ku70 948 GARRKADLIELVKQHFPLPS 967**

HuKu70 Theoretical pI/Mw: 6.23 / 69843.07; LdKu70 Theoretical pI/Mw: 6.43 / 103023.17; 165/609 = 27% identity

**B.** **Amino acids alignment of human Ku80 and *Leishmania donovani* Ku80**

** Hu Ku80 1 MVRSGNKAAVVLCMDVGFTMSNSIPGIESPFEQAKKVITMFVQRQVFAENKDEIALVLFGTDGTDNPL---SGGDQYQNI 77**

** Ld Ku80 1 M----SKGAAVLVLDITLPRA-------AALAEACDLCDRILADKMIYAPSDEVAVILAGTEKSRSALYERSEQARYNHI 69**

** Hu Ku80 78 TVHRHLMLPDFDLLEDIESK-----IQPG--------SQQADFLDALIVSMDVIQHETIGKKFEKRHIEIFTDLSSRFS- 143**

** Ld Ku80 70 TVAADLGPATTLTLAPIAATRAGVAVLPDGATVRRSIAEAYDFIDALQVAVAVLQVRT-SQKRYNRCIYFLTDARHEVRH 148**

** Hu Ku80 144 KSQLDIIIHSLKKCDISLQFFLPFSLGKEDGSGDRGDGPFRLGGHGPSFPLKGITEQQKEGLEIVKMVMISLEGEDGLDE 223**

** Ld Ku80 149 KEDLLSLIGALQRDQVALVVIGFDFQALPAPTDSQGKFNEFTAGEASAW---AALDRKAQNEIILAALCTELGSPSTL-- 223**

** Hu Ku80 224 IYSFSESLRKLCVFK-KIERHSIHWPCRLTIGSNLSIRIAAYKSILQERVKKTWTVVDAKTLKKEDIQKETVYCLNDDDE 302**

** Ld Ku80 224 -VSPAEALASLSLPRCRRIRQQPVLKVALRIGD-VRLATQLFTLTQEERLPSLRRSTQEGVDVVQTIEYVAVGGV-EEQP 300**

** Hu Ku80 303 TEVLKEDIIQGFRYGSDIVPFSKVDEEQMKYKSEGKCFSVLGFCKSSQVQRRFFMGNQVLKVFAARDDEAAAVALSSLIH 382**

** Ld Ku80 301 CALAKEERVEAFFLGADRISCSEADRETMRVKG-PRALEAIGFVGEAEVEPYLLMG-GTRALLPLAGDHAGQRGFNALVD 378**

** Hu Ku80 383 ALDDLDMVAIVRYAYDKRANPQVGVAFPHIKHN---YECLVYVQLPFMEDLRQYMFSSLKNSKKYAPTEAQLNAVDALID 459**

** Ld Ku80 379 AMASSRKAMLVRLVRTADAAPSLCVCFARTAGSSAEQRHLVLAPLPFAEDVRALRFSEYPELQF---SAAEEQLMDELID 455**

** Hu Ku80 460 SMSLAKKDEKTDTLEDLFPTTKIPNPRFQRLFQCLLHRALH--------------PREPLPPI----QQHIWNMLNPPA- 520**

** Ld Ku80 456 GLSVD---------DSVLAPHDTFNPVLQQYYATLRSKLSAMNVSAEKGNAKATSSEAAVPQLLPTLRGTSTDFFAEGSA 526**

** Hu Ku80 521 --EVTTKSQIPLSKIKTLFPLIEAKK-----------------KDQVTAQEIFQDNHEDGPTAKK--------------- 566**

** Ld Ku80 527 VYEAVSAHRAALASCANAFPYEDEVDALLPGSRDAGWKGKPWYQDVATTSSLIDPQAVSPPSASQGSGGAPSTIAAAIAR 606**

** Hu Ku80 567 -------LKTEQGGAHF-----SVSSLAEGSVT-----SVGSVNPAENFRVLVKQKKAS---FEEASNQLINHIEQFLDT 626**

** Ld Ku80 607 GVRCGGEADEASAGSHATSDANSISTAPHDTISGGLSFAITSVDPVGSFSMIVHHPAVTEAQLNKAKDDLSDVIWELLRS 686**

** Hu Ku80 627 N-ETPYFMKSIDCIRAFREEAIKFSEEQRFNNFLKALQEKVEIKQ---LNHFWE-IVVQ--DGITL--ITKEEASGSSVT 697**

** Ld Ku80 687 SIKDAIYRKCMACIMALRQFCVKQDDAAYYNDFL--LKLEVVARQCGRDTDFWAPYVVERKDSANVWPITAQECKSAALP 764**

** Hu Ku80 698 AEEAKKFLAPKDKPSGDTAAVFEEGGDVDDLLDMI- 732**

** Ld Ku80 765 DDTAAKAFLQKDR--FDPAIAFDDTTDDDDWLAEIQ 798**

HuKu80 Theoretical pI/Mw: 5.55 / 82704.54; LdKu80 Theoretical pI/Mw: 4.91 / 85783.08; 158/732 = 22% identity

**C.** **Amino acids alignment of *Leishmania donovani* Ku70 and Ku80**

** Ld Ku70 1 MDEFAEWGEGTTGVVAADSTYTEGLDLDEFREQLWQRNQRDAVVCLVDCNEGMFGVLPAGESAKTTQTGKNGEGAPAHTR 80**

** Ld Ku80 --------------------------------------------------------------------------------**

** Ld Ku70 81 GVTKLALLTIGQGAAMSSGAAGGSKAAVGSSPSFFSMTMQCILALLKEKMMCGSKDVVAIVLYNTRTSAPS-------TG 153**

** Ld Ku80 1 ---------------MSKGAAVLVLDITLPRAAALAEACDLCDRILADKMIYAPSDEVAVILAGTEKSRSALYERSEQAR 65**

** Ld Ku70 154 FRGVYVMQEATRIGTECMQKVEQLEAAGAPGSVAYEEFEARIGHWPTASTSPALAAASSASGRAEPKAALPAALAAFPAF 233**

** Ld Ku80 66 YNHITVAAD---LGPATTLTLA-----------------------PIAATRAGVAVLPDG-------ATVRRSIAE--AY 110**

** Ld Ku70 234 KFSEALWEAQRILLSLRSVQAIRHRRLFVFTNRDDPSGGDAHEWNLCRSRACDLGKEGVVLEVFGFGNAGSGGGPHTSGI 313**

** Ld Ku80 111 DFIDALQVAVAVL-QVRTSQKRYNRCIYFLTDARH----EVRHKEDLLSLIGALQRDQVALVVIGFDFQ----------- 174**

** Ld Ku70 314 SKVAGTVANTSRGELPISLTAPVGADGTGSCSSGSSASPLFAVSNQRGQSLDPAAATTDTTIGQSGSGQSDSPSPAATGF 393**

** Ld Ku80 175 -----------------ALPAPTDSQGKFNEFTAGEASAWAALDRKAQNEIILAALCTEL-------------------- 217**

** Ld Ku70 394 VQDFFWGPLLREMQMAAARFRASGDGDDLVALAEARGEAFVSGGEGAIYMNSGAGALQQLLVSVVRRAHPQRPFRHCLLR 473**

** Ld Ku80 218 -----------------------GSPSTLVSPAEA---------------------LASLSLPRCRRIRQQPVLK----- 248**

** Ld Ku70 474 IGGLSGTSTALSATTAAATARDEEEALRVAAVPRMAVSLYVPLMRARLPQREWLDGRTNRMLRRVVHLNARTTAAGRDSD 553**

** Ld Ku80 249 ------------------------VALRIGDV-RLATQLFTLTQEERLPSLRRS-------------------------- 277**

** Ld Ku70 554 DAGAGNKEGDSGSPTKQLQRLRNEKNCTSESMVRQED----VDPDDLCYYAPVGKERVYFTSEERKRMVEVAATGTEPGF 629**

** Ld Ku80 278 ----------------TQEGVDVVQTIEYVAVGGVEEQPCALAKEERVEAFFLGADRISCSEADR----ETMRVKGPRAL 337**

** Ld Ku70 630 TVLLFKDLVDAVKREHVVRRSSFLHSCVQRGGAHSHRLFVLFVRRLRAKQKVAIAQYCSSTTTAPRLVALVPSPDLTAHP 709**

** Ld Ku80 338 EAIGFVGEA-EVEPYLLMGGTRALLPLA--GDHAGQRGFNALVDAMASSRKAMLVRLVRTADAAPSLCVCFARTAGSS-- 412**

** Ld Ku70 710 EKRDQVPVDGMGLYVVPLPYAEELRAVPELRTCTRVSKHATPVLADSSVDPTHLELAKQVVSALTVS---YQVDAVLNPA 786**

** Ld Ku80 413 -------AEQRHLVLAPLPFAEDVRALRFSEYP------------ELQFSAAEEQLMDELIDGLSVDDSVLAPHDTFNPV 473**

** Ld Ku70 787 LQRQYRKLQELARQFFPLADNPLHPAGGTALAKAGEEGPSTDGAEKTPQELDNTLPDY--EGMQRFAALFQSFNKEVLGK 864**

** Ld Ku80 474 LQQYYATLRSKLSAMNVSAEK------GNAKATSSEAA-----VPQLLPTLRGTSTDFFAEGSAVYEAV--SAHRAALAS 540**

** Ld Ku70 865 DYNAFLY-------CPQPRVAGSATRRPRDSAAGAGAM---EAAGPTAASAEEDACNVSIEELIRRAA-----AENAWDG 929**

** Ld Ku80 541 CANAFPYEDEVDALLPGSRDAGWKGKPWYQDVATTSSLIDPQAVSPPSASQGSGGAPSTIAAAIARGVRCGGEADEASAG 620**

** Ld Ku70 930 LIIPQLKEYLATA--NVSSGGARRKADLIELVKQHFPLPS---------------------------------------- 967**

** Ld Ku80 621 SHATSDANSISTAPHDTISGGLSFAITSVDPVGSFSMIVHHPAVTEAQLNKAKDDLSDVIWELLRSSIKDAIYRKCMACI 700**

** Ld Ku70 --------------------------------------------------------------------------------**

** Ld Ku80 701 MALRQFCVKQDDAAYYNDFLLKLEVVARQCGRDTDFWAPYVVERKDSANVWPITAQECKSAALPDDTAAKAFLQKDRFDP 780**

** Ld Ku70 ------------------**

** Ld Ku80 781 AIAFDDTTDDDDWLAEIQ 798**

141/660 = 21% identity

**D.** **Amino acids alignment of *Leishmania donovani* Ku70 and *Mycobacterium marinum* Ku**

**Score Expect Method Identities Positives Gaps**

**22.3 bits(46) 0.039 Compositional matrix adjust. 48/200(24%) 74/200(37%) 64/200(32%)**

**LdKu70 646 VVRRSSFLHSCVQRGGAHSHRLFVLFVRRLRAKQKVAIAQYCSSTTTAPRLVALVPSPDL 705**

**++ RS FL + S + +VL + L +++AI + + T RL AL**

**MmKu 105 MIDRSYFLEP-----DSKSSKSYVLLAKTLAETERMAIVHFTLRSKT--RLAAL------ 151**

**LdKu70 706 TAHPEKRDQVPVDGMG----LYVVPLPYAEELRAVPELRTCTRVSKHATPVL-ADSSVDP 760**

**V G + V L + +E+R P+ PVL + + P**

**MmKu 152 ----------RVKDFGKREVMVVHTLLWPDEIRD-PDF-----------PVLDKEVEIKP 189**

**LdKu70 761 THLELAKQVVSALTVSYQVDAVLNPALQRQYRKLQELARQFFPLADNPLHPAGGTALAKA 820**

**L++A QVV ++ + D +YR + Q L D L GG A**

**MmKu 190 AELKMAGQVVESMADDFNPD---------RYRDTYQ--EQLQELIDAKLE--GGEAFT-- 234**

**LdKu70 821 GEEGPSTDGAEKTPQELDNT 840**

**AE+ P ELD T**

**MmKu 235 ---------AEEQPAELDET 245**

**E.** **Amino acids alignment of *Leishmania donovani* Ku80 and *Mycobacterium marinum* Ku**

**Score Expect Method Identities Positives Gaps**

**22.3 bits(46) 0.036 Compositional matrix adjust. 28/143(20%) 63/143(44%) 10/143(6%)**

**LdKu80 335 RALEAIGFVGEAEVEPYLLMGGTRALLPLAGDHAGQRGFNALVDAMASSRKAMLVRLVRT 394**

**R +E + FV EV+P ++ R+ L D + + L +A + + +V T**

**MmKu 88 REIEVLEFVPAEEVDPMMI---DRSYF-LEPDSKSSKSYVLLAKTLAETERMAIVHF--T 141**

**LdKu80 395 ADAAPSLCVCFARTAGSSAEQRHLVLAPLPFAEDVRALRFSEY-PELQFSAAEEQLMDEL 453**

**+ L + G ++ +V+ L + +++R F E++ AE ++ ++**

**MmKu 142 LRSKTRLAALRVKDFG---KREVMVVHTLLWPDEIRDPDFPVLDKEVEIKPAELKMAGQV 198**

**LdKu80 454 IDGLSVDDSVLAPHDTFNPVLQQ 476**

**++ ++ D + DT+ LQ+**

**MmKu 199 VESMADDFNPDRYRDTYQEQLQE 221**
