# Supplementary figures and images for: Reconstitution of Mycobacterium marinum Nonhomologous DNA End Joining Pathway in Leishmania
Source: mSphere. 2022 Jun 13;7(3):e00156-22. doi: 10.1128/msphere.00156-22 (PMC9241504; doi:10.1128/msphere.00156-22)

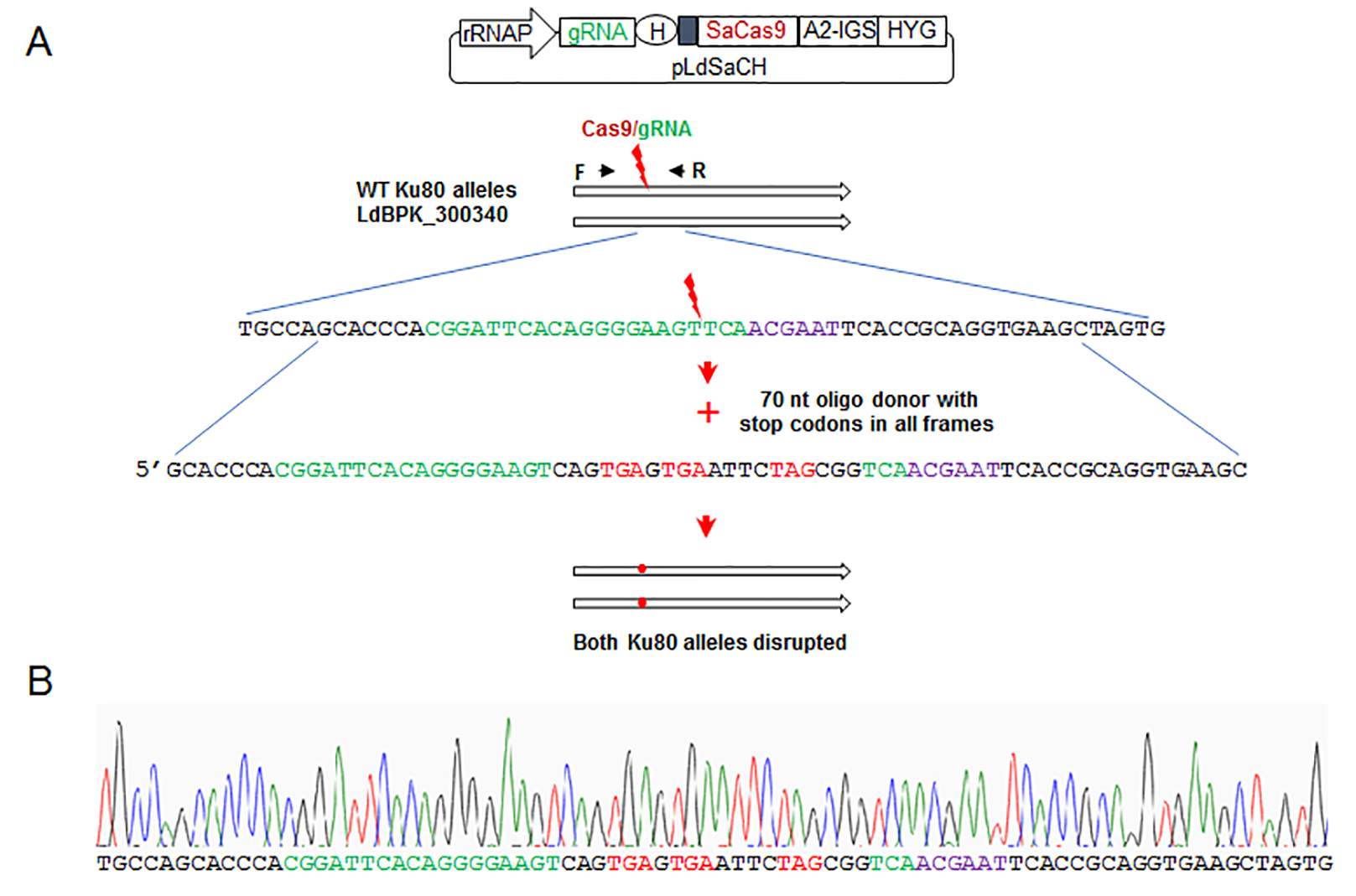

Supplement: FIG S2 [file msphere.00156-22-s0002.tif]
